# Silencing NUDT21 Attenuates the Mesenchymal Identity of Glioblastoma Cells via the NF-κB Pathway

**DOI:** 10.3389/fnmol.2017.00420

**Published:** 2017-12-19

**Authors:** Jia-Cheng Lou, Yu-Long Lan, Jin-Xia Gao, Bin-Bin Ma, Ting Yang, Zhong-Bo Yuan, Hong-Qiang Zhang, Ting-Zhun Zhu, Ning Pan, Song Leng, Gui-Jun Song, Bo Zhang

**Affiliations:** ^1^Department of Neurosurgery, The Second Affiliated Hospital of Dalian Medical University, Dalian, China; ^2^Department of Anesthesiology, The Second Affiliated Hospital of Dalian Medical University, Dalian, China; ^3^Health Management Center, The Second Affiliated Hospital of Dalian Medical University, Dalian, China; ^4^Department of Neurology, The Second Affiliated Hospital of Dalian Medical University, Dalian, China

**Keywords:** NUDT21, glioblastoma, mesenchymal identity, microarray, therapy

## Abstract

The proneural (PN) and mesenchymal (MES) subtypes of glioblastoma multiforme (GBM) are robust and generally consistent with classification schemes. GBMs in the MES subclass are predominantly primary tumors that, compared to PN tumors, exhibit a worse prognosis; thus, understanding the mechanism of MES differentiation may be of great benefit for the treatment of GBM. Nuclear factor kappa B (NF-κB) signaling is critically important in GBM, and activation of NF-κB could induce MES transdifferentiation in GBM, which warrants additional research. *NUDT21* is a newly discovered tumor-associated gene according to our current research. The exact roles of *NUDT21* in cancer incidence have not been elucidated. Here, we report that *NUDT21* expression was upregulated in human glioma tissues and that *NUDT21* promoted glioma cell proliferation, likely through the NF-κB signaling pathway. Gene set enrichment analysis, western blotting, and quantitative real-time reverse transcription polymerase chain reaction confirmed that NF-κB inhibitor zeta (NFKBIZ) was a downstream target affected by *NUDT21* and that the MES identity genes in glioblastoma cells, *CHI3L1* and *FN1*, were also differentially regulated. Our results suggest that *NUDT21* is an upstream regulator of the NF-κB pathway and a potential molecular target for the MES subtype of GBM.

## Introduction

Glioblastoma multiforme (GBM) is the most common malignant glioma in the central nervous system in adults. The median survival of GBM patients is approximately 15 months with treatment, and the 2-year survival rate is only 3% (Stewart, 2002). Most patients with GBM could live for only a few months without treatment (Sowers et al., 2014). The global burden of malignant glioma is expected to rise, partly due to the poor prognosis and aging human population. The dispersed nature and invasive growth of GBMs, together with a high frequency of recurrence, are associated with a poor prognosis (Furnari et al., 2007; Etminan et al., 2011a,b). Thus, new therapy approaches are urgently required. It is known that each GBM tumor is composed of heterogeneous tumor cell populations, including tumor cells with stem cell characteristics, termed glioma initiating cells or glioma stem cells (Hemmati et al., 2003; Singh et al., 2003, 2004). Various studies have shown that the stem cell properties in cancer could contribute to tumor initiation and therapeutic resistance (Bao et al., 2006; Vescovi et al., 2006; Capper et al., 2009; Beier et al., 2011). In various cancers, the transition from the epithelial to the mesenchymal (MES) subtype (epithelial–MES transition; EMT) is associated with advanced malignancy (Nurwidya et al., 2012; Sanchez-Tillo et al., 2012; Shirkoohi, 2013). Interestingly, MES transition also occurs in GBM and can be induced by the master transcription factors (TFs) STAT3, C/EBPb, and TAZ (Carro et al., 2010; Bhat et al., 2011). This EMT-like phenotypic shift in GBM in response to various microenvironmental or external factors is now termed the proneural (PN) to MES transition (PMT; Phillips et al., 2006; Bhat et al., 2013; Mao et al., 2013; Halliday et al., 2014). PN and MES are two robust subtypes of GBM that are generally consistent with the classification schemes. GBMs in the MES subclass are predominantly primary tumors and exhibit a worse prognosis compared to PN tumors (Pelloski et al., 2005; Phillips et al., 2006; Colman et al., 2010). Therefore, determining the precise mechanism of MES differentiation will have great implications for the treatment of GBM. Currently, more efforts should be directed toward clarifying the molecular mechanisms that control the phenotypic shift of the two subtypes of GBM.

It has recently been demonstrated that nuclear factor kappa B (NF-κB) is a master regulator that drives MES transdifferentiation (Bhat et al., 2013). The NF-κB proteins are a family of TFs mediating immune and inflammatory responses (Robe et al., 2004; Angileri et al., 2008; Korkolopoulou et al., 2008). NF-κB signaling is critically important during glioma development and progression (Basseres and Baldwin, 2006; Hoffmann and Baltimore, 2006; Karin, 2006). Various studies have found that levels of NF-κB activity in GBM tissues, as determined by serine phosphorylation, are much higher than in normal tissues (Wang et al., 2004; Nozell et al., 2008) and correspond with an increasing grade of astrocytic tumors (Angileri et al., 2008; Korkolopoulou et al., 2008). Using specific inhibitors to inhibit the activation of NF-κB could inhibit glioma growth; however, the precise mechanism of NF-κB activation in GBMs is currently poorly understood. NF-κB inhibitor zeta (NFKBIZ) is a protein encoded by the *NFKBIZ* gene in humans (Eto et al., 2003; Totzke et al., 2006). Unlike various other typical NF-κB proteins, NFKBIZ is not rapidly degraded but, rather, stably accumulates in the nucleus where it inhibits NF-κB activity (Totzke et al., 2006). Importantly, inhibiting the activation of NF-κB may be an effective alternative therapy for inhibiting GBM growth (Robe et al., 2004).

Located in the last or 3′-most exons, alternative poly(A) sites could lead to the production of mRNAs with variable 3′-untranslated regions, resulting in protein products that vary at the C-terminus (Elkon et al., 2013). *NUDT21*, a tumor-associated gene, has been studied for many years, and the protein encoded by this gene, the Cleavage Factor Im (*CFIm25*), is one subunit of a cleavage factor required for 3′-RNA cleavage and polyadenylation processing (Masamha et al., 2014). Previous studies found that CFIm is a heterodimer consisting of a 25-kDa protein (NUDT21/CPSF5) and a larger subunit. A complex consisting of NUDT21 and a 68-kDa subunit protein (CPSF6) are sufficient for RNA binding (Dettwiler et al., 2004), and this heterodimer reconstitutes CFIm processing activity (Ruegsegger et al., 1996). Additional larger subunits (59 and 72 kDa) also have been identified (Dettwiler et al., 2004; Venkataraman et al., 2005), but their significance remains uncertain. Besides, this heterodimer also appear to act in 3′-end processing of *NEAT1_1*, which is a specific long non-coding RNA, including two isoforms *NEAT1_1* and *NEAT1_2* (Naganuma et al., 2012). The authors observed that the RNAi of *NUDT21* or *CPSF6* markedly diminished *NEAT1_1* levels and simultaneously increased the *NEAT1_2* level. Furthermore, it has been reported currently that alterations in the *NUDT21* gene cause changes in the level of MeCP2 protein in cells and leads to neuropsychiatric diseases (Gennarino et al., 2015).

*NUDT21* plays a pivotal role in 3′-end processing and might be indispensable for elucidating the fundamental mechanisms regarding regulating alternative poly(A) site selection, thus controlling glioma malignancy. The exact roles of *NUDT21* in GBM and various other cancers have not been elucidated, thus more research is warranted. Specifically, it remains to be determined whether *NUDT21* plays a role in GBM pathophysiology. In the present study, driven by the fact that *NUDT21* is a promising candidate from gene screening, we sought to examine its potential roles in glioma incidence, tumor growth and MES transdifferentiation. Interestingly, the association between *NUDT21* and the NF-κB signaling pathway has been further elucidated. Here, we utilized *in vivo* and *in vitro* techniques to identify the precise mechanisms underlying the role of *NUDT21* in GBM promotion and MES identity determination. Our results indicate that targeting the *NUDT21*-induced NF-κB signaling axis may be a therapeutic strategy for the treatment of GBM patients with an MES signature.

## Materials and Methods

### Cancer Patient Samples

Samples were harvested from 15 fresh matched pairs of tissues samples isolated from GBM patients who were hospitalized in The Second Affiliated Hospital of Dalian Medical University between September 2014 and December 2016 (**Supplementary Table S2**). Tumoral tissues in addition to their adjacent non-cancerous tissues (ANCTs) were obtained from all patients during surgery under the conventions of the Ethics Committee. ANCT was defined as the normal breast tissue diagnosed by the pathologists through H&E staining. Written informed consent was obtained from all patients. The Ethics Committee of The Second Affiliated Hospital of Dalian Medical University approved the study protocol, and all experiments were performed in accordance with the approved guidelines.

### Cell Culture

Human U87MG (U87) and U251 cell lines were obtained from American Type Culture Collection (Manassas, VA, United States). Cells were maintained in DMEM supplemented with 10% fetal bovine serum (FBS). All cell cultures were maintained at 37°C in a humidified atmosphere containing 5% CO_2_.

### NUDT21 Knockdown Using shRNAs

The lentiviral vectors were purchased from Shanghai Genechem Company Ltd., China. A non-silencing shRNA (5′-GCCTAACTGTGTCAGAAGGAA-3′) was used as the negative control (shCtrl). The siRNA sequence targeting the *NUDT21* gene was 5′-ACCTCCTCAGTATCCATAT-3′. Cells were seeded into a six-well plate (∼5 × 10^4^ cells per well) and incubated at 37°C with 5% CO_2_ until they reached ∼30% confluence before transfection.

### Cell Viability Assay

Cell proliferation was determined by MTT (Roche Diagnosis, Indianapolis, IN, United States) assay. Cells were plated at a certain density (1 × 10^3^ cells per well) in 96-well plates. Cells were then incubated in complete media for another 5 days. After 5 days incubation, cell growth was measured. The effect of *NUDT21* knockdown on cell viability was assessed as the percent cell viability compared with the untreated control group, which was assigned 100% viability. The optical density (OD) values were determined (wavelength: 490 nm). All experiments were performed in triplicate.

### Celigo Assay

After achieving the logarithmic growth phase, U87MG or U251 cells were digested with trypsin, resuspended in standard medium, and then seeded into 96-well plates at a density of 2,000 cells/well. The number of green fluorescent protein fluorescence-positive cells was counted using a Cellomics Array Scan High Contents Screening Reader on five consecutive days.

### Cell Cycle Analysis

Cells were plated in six-well plates for 24 h. After the G1/GO synchronization by FBS deprivation for 48 h, cells were incubated in DMEM supplemented with 10% FBS again for another 48 h. Then cells were collected and washed once with cold PBS, then stained with a propidium iodide (PI) solution (50 mg/mL PI and 0.5 mg/mL RNase in TBST buffer) for 15 min at 4°C. Cell cycle distribution was measured using a FACS Accuri C6 flow cytometer (Genetimes Technology Inc.).

### Western Blot Analysis

Cell total proteins were collected using RIPA lysis buffer containing phosphatase and protease inhibitors (Roche, Switzerland) in accordance with the manufacturer’s instructions. Glioma and normal tissues were lysed in lysis buffer with shaking at 4°C for 30 min. Proteins were quantified by a bicinchoninic acid assay protein assay kit. Proteins were separated by SDS-PAGE, and then transferred to PVDF membranes, blocked with 5% fat-free dry milk or 5% BSA in TBST and immunoblotted with primary antibodies at 4°C overnight. The following day, the membranes were incubated with second antibodies at room temperature for 2 h. The protein bands were detected by enhanced chemiluminescence. The NFKBIZ antibody was purchased from Proteintech. The fibronectin-1 (FN1) and chitinase-3-like protein 1 (CHI3L1) antibodies were purchased from Abcam.

### Quantitative Real-Time Reverse Transcription Polymerase Chain Reaction

Total RNA was obtained using the TRIzol one-step RNA isolation kit (TaKaRa Bio, Dalian, China). Specific cDNA were synthesized from total RNA using PrimeScript RT Reagent Kit (TaKaRa Bio, Dalian, China) according to the manufacturer’s instructions. The relative mRNA expression of each gene was normalized to glyceraldehyde 3-phosphate dehydrogenase (GAPDH) RNA levels and was analyzed using the 2^-ΔΔ*C*_T_^ method. The primers were synthesized by Invitrogen (Shanghai, China). The primers were sense 5′-GGTCACTCAGTTCGGCAACAA-3′, antisense 5′-CTCATGCGCTGAAATCTGGC-3′ for NUDT21; sense 5′-CAAAGGCTTACAATGGCAAC-3′, antisense 5′-CATCGGGAACCAAATGCAC-3′ for NFKBIZ; and sense 5′-TGACTTCAACAGCGACACCCA-3′, antisense 5′-CACCCTGTTGCTGTAGCCAAA-3′ for GAPDH.

### Microarray Gene Expression Analysis

Total RNA was extracted from U87MG cells after transfection with shNUDT21, and 50–500 ng of RNA was used to generate biotin-modified amplified RNA (aRNA) using a GeneChip 3′ IVT Express Kit (Affymetrix, United States). Reverse transcription was performed using a T7 oligo (dT) primer, and a first-strand IVT Labeling Master Mix was used to produce multiple copies of biotin-modified aRNA. The aRNA was then purified and quantified. After fragmentation, the aRNA was hybridized to the GeneChip PrimeView Human gene expression array cartridge (Affymetrix, United States). After hybridization, the chips were stained with phycoerythrin and washed in a GeneChip Fluidics Station 450. The microarray signals were scanned and analyzed using a GeneChip Array Scanner 3000 7G.

Gene signatures were compared between the shCtrl and shNUDT21 cells. To identify pathways commonly deregulated in the shNUDT21 U87MG cells, enrichment of the differentially expressed gene signatures in human pathways was evaluated by gene set enrichment analysis (GSEA; Subramanian et al., 2005) using pathways collected in the hallmark and c2 curated gene sets^1^ with 1,000 gene label permutations (gene sets). Significantly enriched gene sets, defined by nominal *p* < 0.05, were compared between shCtrl and shNUDT21 gene signatures.

### Animal Studies

All the animals were maintained in the SPF Laboratory Animal Center at Dalian Medical University, which was also the site at which all the animal experiments were performed. Female nu/nu mice (4–6 weeks old) were used for these experiments. To evaluate the effect of *NUDT21* knockdown in a human U87MG xenograft glioma mouse model, we subcutaneously injected U87MG cells (2 × 10^6^ in 100 μl PBS) near the axillary fossae of the nude mice using a 27-gauge needle. The tumor cell-inoculated mice were then randomly divided into two groups, each of which contained five mice. The tumors were measured with a caliper every day, and tumor volumes were calculated using the following formula: *V* = 1/2 (width 2 × length). Body weights were also recorded. On day 5 after tumor cell inoculation, all the experimental mice were euthanized with ether anesthesia, and their total tumor weights were measured.

All animal maintenance and procedures were carried out in strict accordance with the recommendations established by the Animal Care and Ethics Committee of Dalian Medical University as well as the guidelines by the United States National Institutes of Health Guide for the Care and Use of Laboratory Animals. The protocol was approved by the Animal Care and Ethics Committee of Dalian Medical University. In the animal study, all efforts were made to minimize the suffering of the mice. All mice were humanely sacrificed under ether anesthesia inhalation before death.

### Statistical Analysis

Student’s *t*-test (two-tailed), *t*-test with Welch’s correction, *F*-test were performed to analyze the data using GraphPad Prism 6.0 software. The concrete methods of *t*-test analysis in current study are as follows: the data of two groups for comparison were analyzed by *F*-test firstly (homogeneity test of variance): If the value of *F*-test >0.05, the value of *t*-test was obtained according to heteroscedasticity double sample test. If the value of *F*-test <0.05, the value of *t*-test was obtained according to the heteroscedasticity double sample test; the value of *t*-test <0.05 indicated that there was significant difference between the two experimental groups, and the value of *t*-test >0.05 indicated that there was no significant difference between the two experimental groups. Two-way analysis of variance (ANOVA), followed by a Bonferroni’s test for multiple comparisons, were performed to analyze the data for **Figures 2C,D,F**, **3A** and **Supplementary Figures S1B,E**, **S2A**. While one-way ANOVA, followed by a Tukey’s post-test for multiple comparisons, were performed to analyze the data for **Figure 5B**. For most of the *in vitro* and animal experiments, Student’s *t*-tests were used to calculate the *p*-value. *p*-Values less than 0.05 were considered statistically significant. ns, significant; ^∗^*p* < 0.05; ^∗∗^*p* < 0.01; ^∗∗∗^*p* < 0.001; ^∗∗∗∗^*p* < 0.0001.

## Results

### *NUDT21* Expression Is Upregulated in Human GBM Tissues

**FIGURE 1:**
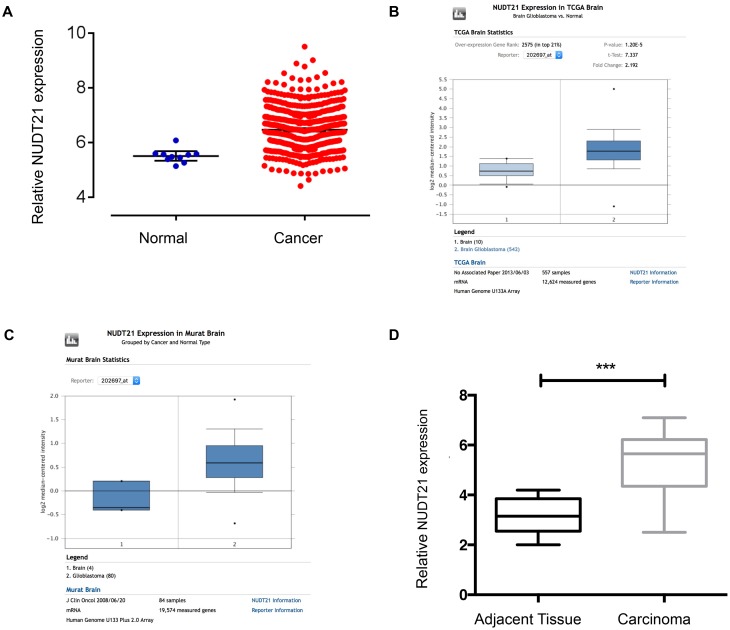
*NUDT21* expression is upregulated in human GBM tissues. **(A)** The correlation of *NUDT21* RNA expression levels with the incidence of GBM (WHO IV) was examined. We selected glioma-related keywords in the data download page and downloaded TCGA Level-3 documents of HT_HG-U133A type in Exp-Gene. According to barcode information of selected samples, 539 GBM (WHO IV) samples and 10 normal brain samples were selected. We then used Affy and Limma packages to normalize and exert *t*-test using R programming language. After selection based on *p*-value <0.05 and |logFC|≥ 1 (with 2 as the base), we finally got that FC = 2.0001, *p* = 4.66E-05, FDR = 1.44E-04. **(B)** *NUDT21* expression in TCGA Brain dataset was analyzed by Oncomine website tools. *NUDT21* was significantly (*p* < 0.0001) upregulated in GBM tissues (*n* = 542) compared with normal brain tissues (*n* = 10). **(C)** *NUDT21* expression in Murat Brain dataset was analyzed by Oncomine website tools. *NUDT21* was significantly (*p* = 0.003) upregulated in GBM tissues (*n* = 80) compared with normal brain tissues (*n* = 4). **(D)** *NUDT21* expression was examined using RT-qPCR in 15 pairs of GBM tissues in patients compared with corresponding non-tumor tissues, and the *NUDT21* expression was significantly increased in GBM tissues. Error bars, standard error. ^∗∗∗^*p* < 0.001.

To examine the *NUDT21* expression level, we analyzed TCGA Level-3 expression levels in GBM tissues compared with normal tissues and found that *NUDT21* was significantly upregulated in GBM tissues (**Figure 1A**). Then, we re-analyzed *NUDT21* expression in GBM or normal tissue based on public data on the Oncomine website^2^ and found that *NUDT21* expression was also significantly increased in GBMs of two different datasets (**Figures 1B,C**). To confirm this result, we examined *NUDT21* expression using quantitative real-time reverse transcription polymerase chain reaction (RT-qPCR) in 15 pairs of GBM tissues and corresponding non-tumor tissues (**Figure 1D**). All these indicated that upregulation of *NUDT21* may be important for GBM pathogenesis.

### Functional Loss of *NUDT21* Impacts GBM Cell Proliferation *in Vitro*

**FIGURE 2:**
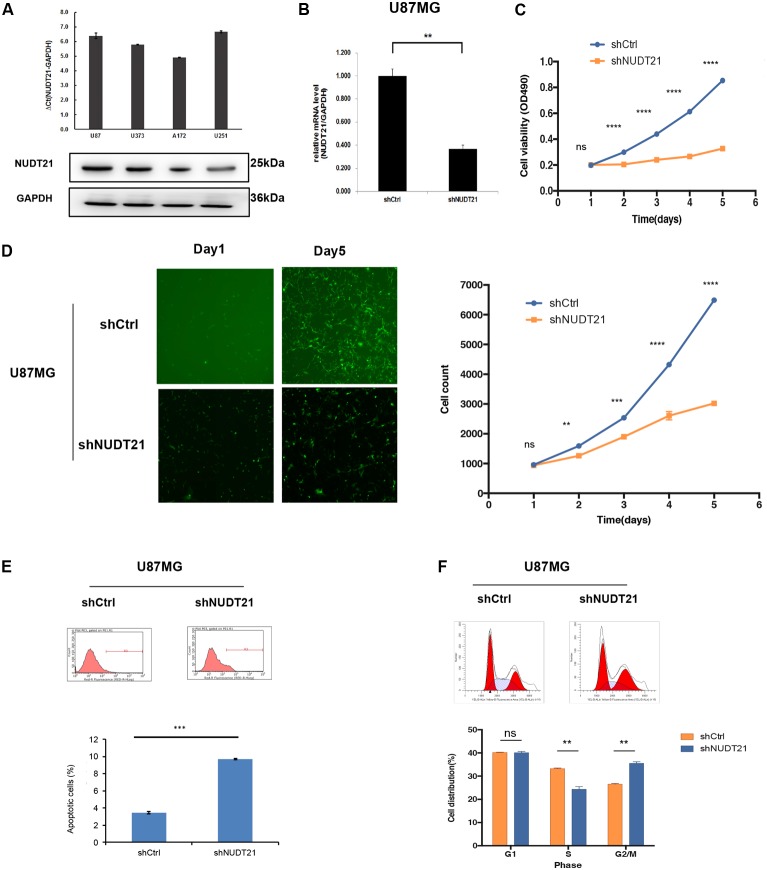
Knocking down *NUDT21* suppresses glioblastoma cell proliferation. **(A)** The results of RT-qPCR indicated that all four glioblastoma cell lines could be of high-expression of *NUDT21* [ΔCt = Ct value of NUDT21 – Ct value of GAPDH (control gene), mean ± SD, *n* = 3]. The U87MG and U251 cell lines were selected for further study to investigate the biological role of *NUDT21* in GBM cells. **(B)** *NUDT21* knockdown was accomplished using shRNAs in glioma cells (mean ± SD, *n* = 3, ^∗∗^*p* < 0.01). **(C)** MTT assay showed that, compared with the cell proliferation in the control cells, *NUDT21* knockdown significantly decreased cell proliferation in U87MG cells. The OD values were measured at indicated days (mean ± SD, *n* = 3, ns, significant; ^∗∗∗∗^*p* < 0.0001). **(D)** Celigo assay was performed to detect the cell viability over 5 days after transfection. The colony numbers of U87MG cells transfected with shCtrl were evidently higher than those transfected with shNUDT21 (mean ± SD, *n* = 3, ns, significant; ^∗∗^*p* < 0.01; ^∗∗∗^*p* < 0.001; ^∗∗∗∗^*p* < 0.0001). **(E)** To further determine the physiological role of *NUDT21* in cell growth, U87MG cells were transfected with shNUDT21. After 48 h, the apoptotic rates of cells were detected by flow cytometry (mean ± SD, *n* = 3, ^∗∗∗^*p* < 0.001). **(F)** Flow cytometry analysis was performed to further examine the effect of *NUDT21* on the proliferation and altered cell cycle progression of GBM cells. The bar chart represented the percentage of cells in G0/G1, S, or G2/M phase (mean ± SD, *n* = 3, ns, significant; ^∗∗^*p* < 0.01 versus shCtrl group at indicated phase).

The results of RT-qPCR indicated that all four glioblastoma cell lines could be of high-expression of *NUDT21*. To investigate the biological consequence of the loss of *NUDT21* in GBM cells, the U87MG and U251 cell lines were selected for further study (**Figure 2A**). First, *NUDT21* knockdown was performed using shRNAs (**Figure 2B**). Next, the MTT assay indicated that, compared with the cell proliferation in control cells, *NUDT21* knockdown significantly decreased cell proliferation in U87MG cells. The OD values of three accessory holes were measured, followed by statistically analysis (*t*-test) between shNUDT21 group and control group at indicated days (**Figure 2C**). Next, the Celigo assay was performed to detect cell viability, and *t*-test was exerted to confirm the statistical significance of differences between shNUDT21 group and control group at indicated days. The colony numbers of U87MG cells transfected with shCtrl were significantly higher than those transfected with shNUDT21 (**Figure 2D**). These results indicated that the functional loss of *NUDT21* inhibits GBM cell proliferation. U87MG cells were transfected with shNUDT21 to further elucidate the physiological role of *NUDT21* in glioma cell growth. After 48 h, cell apoptosis was analyzed using flow cytometry. Our experiments confirmed that transfection of shNUDT21 enhanced apoptosis (**Figure 2E**). Besides, flow cytometry analysis was performed to further examine whether *NUDT21* could affect the proliferation of GBM cells by altering cell cycle progression. Above all, shNUDT21 transfected into U87MG cells could block cell cycle progression (**Figure 2F**). The bar chart represented the percentage of cells in G0/G1, S, or G2/M phase, as indicated. And the results indicated that cells were arrested in the G2/M phase, the percentage of cells in the S phase decreased, while the G1 phase did not change significantly. Then we exerted *t*-test to further confirm the significance of differences between groups. The results of other GBM cell line, U251, confirmed those observed in U87MG cells (**Supplementary Figure S1**). Above all, these results suggested that the functional loss of *NUDT21* may inhibit cell proliferation, block cell cycle progression and promote cell apoptosis.

### Functional Loss of *NUDT21* Inhibits GBM Tumorigenesis *in Vivo*

**FIGURE 3:**
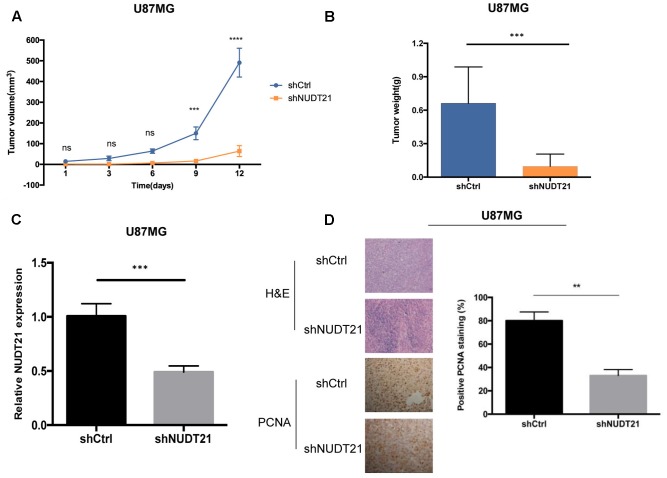
*NUDT21* knockdown inhibits xenograft tumorigenicity *in vivo* in nude mice. U87MG cells and U251 cells (see **Supplementary Figure S2**) were injected into the flanks of the nude mice. **(A)** Tumor volumes were measured on the indicated days (mean ± SD, *n* = 10, ns, significant; ^∗∗∗^*p* < 0.001, ^∗∗∗∗^*p* < 0.0001 versus shCtrl group at indicated days). **(B)** Tumor weights were measured 16 days after injection (mean ± SD, *n* = 10, ^∗∗∗^*p* < 0.001). **(C)** RT-qPCR analysis of *NUDT21* expression in tumor tissues transfected with shNUDT21 or shCtrl. The relative levels of *NUDT21* mRNA were normalized to GAPDH and compared with the expression in tissues transfected with shCtrl (mean ± SD, *n* = 3, ^∗∗∗^*p* < 0.001). **(D)** Immunohistochemistry analysis of *NUDT21* expression in tumors developed from shNUDT21-transfected U87MG cells and tumors formed by shCtrl-transfected U87MG cells. Semi-quantitative analysis of the stained sections was performed using light microscopy to calculate the percentage of positive PCNA staining (mean ± SD, *n* = 3, ^∗∗^*p* < 0.001).

To examine whether the *NUDT21* expression level could affect tumorigenesis, shCtrl/shNUDT21-transfected U87MG cells were inoculated into nude mice. The average tumor volume was measured in each group, followed by statistically analysis (*t*-test) between shNUDT21 group and control group at indicated days. It could be indicated that the tumor growth rate was significantly slower in the shNUDT21 group than that in the control group (**Figure 3A**). Up to 16 days after injection, the average tumor weight in the shNUDT21 group was markedly lower than that in the shCtrl group (**Figure 3B**). Then we exerted RT-qPCR analysis, to examine the *NUDT21* expression level in tumor tissues (**Figure 3C**). Furthermore, the tumors developed from shNUDT21-transfected U87MG cells were also found to display lower proliferating cell nuclear antigen (PCNA) staining compared to the tumors formed by shCtrl-transfected U87MG cells, as detected by immunohistochemistry analysis (**Figure 3D**). Besides, the data of U251 cells further determined the role of *NUDT21* expression in GBM tumorigenesis (**Supplementary Figure S2**).

### Silencing *NUDT21* May Exert a Critical Effect on MES Identity in GBM Cells via the NF-κB Pathway

Abnormal activation of the MES phenotype in GBM is associated with an increase in cellular motility, glycolysis and genes related to inflammatory properties (Zhong et al., 2010). In order to elucidate the molecular mechanisms underlying the role of *NUDT21* in GBM cell phenotypes, we performed gene microarray analysis to compare gene expression of shCtrl-transfected versus shNUDT21-transfected U87MG cells. Differentially expressed genes with more than 1.5-fold change in could be identified. The results demonstrated that 293 and 457 genes were upregulated and downregulated in the shNUDT21-transfected cells, respectively (**Supplementary Table S1**).

**FIGURE 4:**
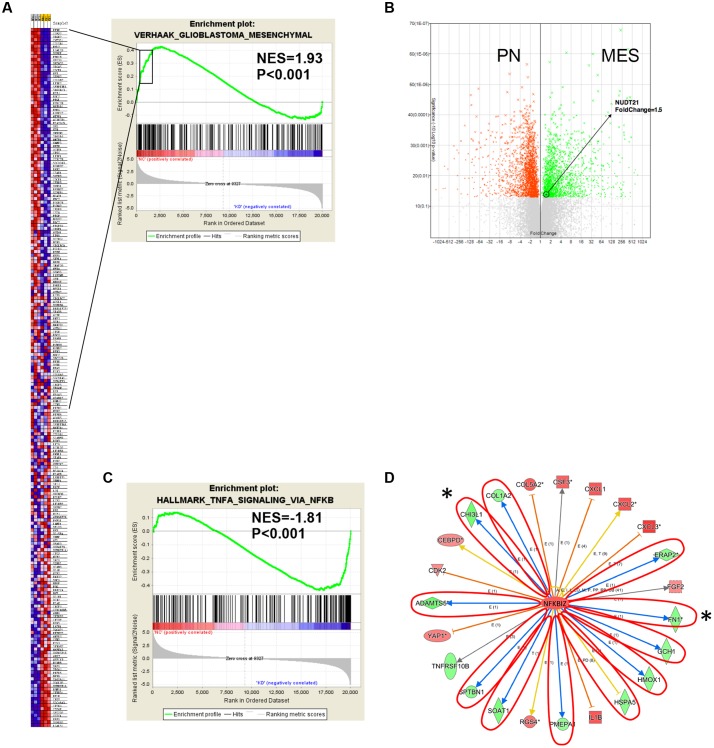
Gene expression profile analysis revealed the downstream targets affected by *NUDT21*. **(A)** The *NUDT21* Knockdown caused a significant reduction in the mesenchymal (MES) signature. **(B)** Other microarray data showed that *NUDT21* expression was significantly upregulated with 1.5-fold change in the MES group of GBM. **(C)** Genes affected by *NUDT21* silencing in GBM cells were enriched with the NF-κB pathway signature. **(D)** The knowledge-based interactome surrounding the regulation of NFKBIZ was examined using Ingenuity Pathway Analysis and overlaid with the microarray data results with a 1.5-fold change cut-off. The blue arrows indicate inhibition; the orange arrows indicate activation; the yellow arrows indicate that the findings are inconsistent with state of the downstream molecule; the gray arrows indicate that the effect is not predicted. ^∗^Means remarkable genes.

Next, we conducted GSEA (Subramanian et al., 2005) to determine whether particular gene sets were enriched in differentially expressed genes regulated by *NUDT21*. Several gene set classifications, as annotated by the collections of GSEA, were significantly enriched. Interestingly, we found that the knockdown *NUDT21* signature was significantly reduced in cells with the MES signature (**Figure 4A**). In addition, other microarray data (Kim et al., 2016) also showed that *NUDT21* expression was significant upregulated with 1.5-fold change in the GBM cells classified as MES (**Figure 4B**).

Recently, studies have demonstrated that NF-κB is a master regulator of MES transdifferentiation. Our GSEA results showed that genes affected by *NUDT21* silencing in GBM cells had an enriched NF-κB pathway signature (**Figure 4C**). Thus, we decided to focus on the differential expression of the NF-κB signaling pathway. NFKBIZ, as an inhibitor of NF-κB activity, was identified as being upregulated by 1.5-fold after *NUDT21* silencing. To further determine targets regulated by NFKBIZ, we examined the knowledge-based interactome surrounding the regulation of NFKBIZ using the Ingenuity Pathway Analysis, and then we overlaid the results with the microarray data that exhibited a 1.5-fold change cut-off. Several targets reported to be involved in the MES identity in GBM cells were differentially regulated, including *CHI3L1* and *FN1* (**Figure 4D**). These data indicate *NUDT21* as an upstream regulator of NF-κB signaling that could affect MES identity in GBM cells.

### Silencing *NUDT21* Could Increase *NFKBIZ* Expression and Downregulate the MES Identity Genes *CHI3L1* and *FN1*

**FIGURE 5:**
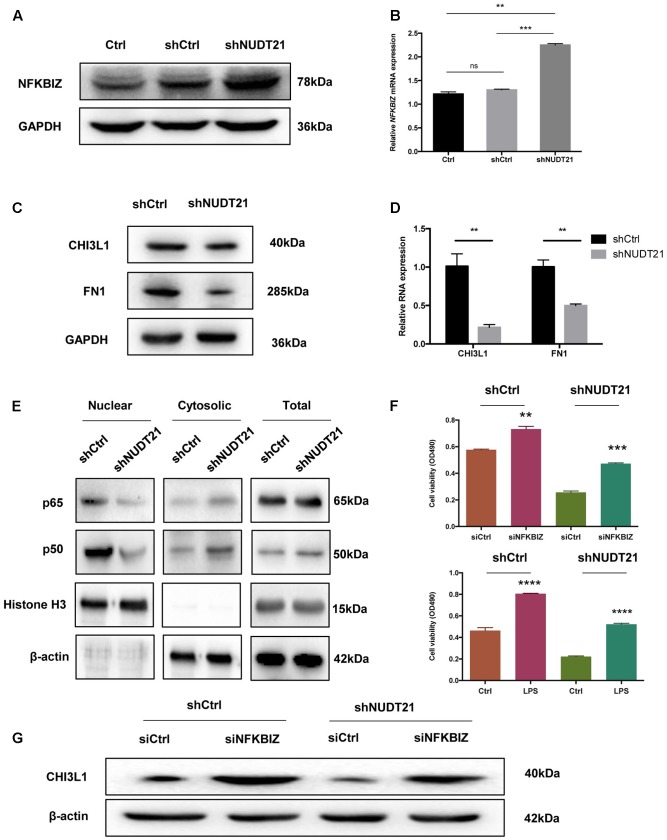
The results of the microarray data regarding the roles of NFKBIZ, NF-κB, and MES identity genes were further validated. **(A,B)** RT-qPCR and western blotting showed that NFKBIZ was upregulated in *NUDT21*-silenced U87MG cells compared with shCtrl-transduced cells (mean ± SD, *n* = 3, ^∗∗^*p* < 0.001 versus Ctrl or shCtrl). **(C,D)** RT-qPCR and western blotting showed that *NUDT21* knockdown in U87MG cells decreased the expression of the MES identity genes CHI3L1 and FN1 (mean ± SD, *n* = 3, ^∗∗^*p* < 0.001 versus Ctrl or shCtrl). **(E)** Western blotting showed that downstream NF-κB expression was affected. Knocking down *NUDT21* inhibited NF-κB translocation from the cytoplasm into the nucleus in glioma cells. Nuclear and cytosolic NF-κB expression levels were analyzed by western blotting, and a quantitative analysis was performed to compare p65 and p50 translocation levels between the different treatment groups. **(F)** The roles of NFKBIZ and NF-κB in the shNUDT21-mediated downregulation of cell proliferation have also been determined. After 48 h, the OD values were measured (mean ± SD, *n* = 3, ^∗∗^*p* < 0.001, ^∗∗∗^*p* < 0.001, and ^∗∗∗∗^*p* < 0.0001 versus siCtrl or Ctrl). **(G)** Western blotting showed that siCHI3L1 reversed the decreased expression by *NUDT21* knockdown. Error bars, standard error.

To validate the results of the microarray analysis, RT-qPCR and western blotting were conducted, and both showed that, compared with NFKBIZ in shCtrl-transfected cells, NFKBIZ was upregulated in *NUDT21*-silenced U87MG cells. And the result of *t*-test further confirmed the statistical significance of difference between Ctrl group and shNUDT21 group or between shCtrl group and shNUDT21 group (**Figures 5A,B**). To confirm whether *NUDT21* regulates *CHI3L1* and *FN1* expression, we knocked down the expression of *NUDT21* in U87MG using shNUDT21. The results indicated that knockdown of *NUDT21* in U87MG cells decreased the expression of MES identity genes *CHI3L1* and *FN1* (**Figures 5C,D**). In addition, since NFKBIZ regulates NF-κB signaling by acting as an inhibitor of NF-κB (Yamazaki et al., 2001), we further examined the downstream NF-κB expression (**Figure 5E**). NFKBIZ could bind to the promoter region of secondary response genes by forming a complex with either p50 homodimers or p50–p65 heterodimers of NF-κB to exert transcriptional regulation. Although NFKBIZ has demonstrated an inhibitory role (Yamazaki et al., 2001), its function as a transcriptional activator dominates. Besides, the role of NFKBIZ and NF-κB in the shNUDT21-mediated downregulation of cell proliferation have also been determined (**Figure 5F**). Cell proliferation assay indicated that *NFKBIZ* silencing could reverse the effect of *NUDT21* in human glioma, and NF-κB nuclear translocation induced by LPS could also reverse the anticancer effect of *NUDT21* knockdown. All these indicated that both NFKBIZ and NF-κB played a role in the shNUDT21-mediated reduced proliferation. Furthermore, to further determine the role of *NUDT21* in the NF-κB signaling, the regulating effect of NFKBIZ on the *NUDT21*-mediated downstream factor CHI3L1 was detected. Western blotting showed that siCHI3L1 reversed the decreased expression by *NUDT21* knockdown (**Figure 5G**).

## Discussion

*NUDT21* is a newly discovered gene that could be of great significance in glioma malignancy. However, more efforts should be directed toward clarifying the functions of this gene, and additional proteins encoded by *NUDT21* may be essential for glioma survival and malignancy. For the first time, we show that *NUDT21* can promote glioma cell proliferation by activating NF-κB signaling. In this study, we used human GBM cells and analyzed the expression of *NUDT21* to elucidate the functional significance of *NUDT21* in the incidence of GBM. Compared with *NUDT21* expression in normal counterparts, *NUDT21* expression levels in patient-derived GBM cells were significantly upregulated. RT-qPCR further confirmed this observation. Thus, *NUDT21* could play an important role in GBM malignancy. Additional studies also indicated that, compared with the cell proliferation in the control cells, *NUDT21* knockdown significantly increased cell proliferation, both in U87MG and U251 cell lines, and the effect of *NUDT21* on GBM cell proliferation was exerted by altering cell cycle progression and enhancing apoptosis. Importantly, functional loss of *NUDT21* also inhibited GBM tumorigenesis *in vivo*, according to the observed growth inhibition of xenograft tumors in mice.

It is known that gene expression profiling of GBM has consistently shown the PN and MES subtypes (Bhat et al., 2011). MES GBMs exhibit a high degree of macrophage/microglial infiltration, and macrophages and microglia may provide extrinsic signals to promote PMT through NF-κB activation (Bhat et al., 2013). It is thus important to further examine the precise mechanisms and influencing microenvironmental factors those could cooperate to sustain the MES identity in GBM tumors. Currently, whether persistent MES identity or MES phenotype shift influenced by *NUDT21* could promote tumor growth is unknown that warrant more research regarding molecular and phenotypic characterization of these tumors. To clarify the molecular mechanisms by which *NUDT21* contributes to the phenotypes of GBM cells, microarray analysis was conducted to explore the gene expression differences in U87MG shCtrl versus U87MG shNUDT21 cells. Differentially expressed genes with more than 1.5-fold change were identified. Several gene set classifications, as annotated by the collections of GSEA, were significantly enriched, and interestingly, we found that the *NUDT21* knockdown signature was related to a significant reduction in the MES signature. Currently, more efforts should be directed toward clarifying the precise mechanisms of this interaction.

A regulatory role has been identified in this study for *NUDT21* in NF-κB signaling in GBM cells, through affecting the MES identity. Intriguingly, NFKBIZ, as an inhibitor of NF-κB activity, was identified as being upregulated by 1.5-fold after *NUDT21* silencing. However, the precise mechanisms regarding the regulatory effect of *NUDT21* on NFKBIZ needs further clarification. In addition, several targets that were reported to be involved in MES identity in GBM cells, including *CHI3L1* and *FN1*, were differentially regulated. These data implicate *NUDT21* as the regulator of NF-κB signaling, which may affect the MES identity of GBM cells. Ultimately, *NUDT21* was determined as a novel candidate for identifying malignant gliomas. We also elucidated a mechanism of pathogenesis by activating the NF-κB pathway via altering *NFKBIZ* activation and various other targets that were reported to be involved in the MES identity of GBM cells.

Although NF-κB is a potentially promising candidate for MES GBM treatment, it should not be ignored that excessive NF-κB inhibition is probably detrimental because of its suppression of innate immunity (Greten et al., 2007; Baud and Karin, 2009). Currently, NF-κB-targeting cancer therapeutics have not been established and the *NUDT21*-driven NF-κB signaling axis might be a therapeutic target for MES GBMs. Furthermore, since *NUDT21* is hardly expressed in normal progenitors and normal brain cells, the toxicity of specific *NUDT21* silencers should be modest.

## Conclusion

We found that *NUDT21* could promote both *in vivo* and *in vitro* GBM cell proliferation, and our study implicates a critical role for *NUDT21* in regulating the MES identity in GBM and suggests *NUDT21* as an attractive therapeutic target for MES GBM treatment. The poorly characterized *NUDT21* gene is abundantly expressed GBM tissue. Given the great significance of NF-κB signaling in cancer, our findings may not only help to better understand the molecular mechanisms that maintain the characteristics of GBM stem cells, but also identify a molecular therapeutic target for GBM treatment.

In addition, identification of *NUDT21*-specific inhibitors can potentially advance the development of molecular-targeted therapeutics for various cancers like GBM. *NUDT21* may target NFKBIZ signaling and various downstream targets that were found to be involved in the MES identity of GBM cells. These findings provide strong evidence for *NUDT21* to be a novel target in malignant glioma treatment.

## Author Contributions

J-CL, Y-LL, and BZ conceived and designed the experiments. J-CL, Y-LL, and J-XG performed all experiments except for the following: B-BM performed IF staining, H-QZ, Z-BY, SL, and G-JS generated patient-derived lines, B-BM and T-ZZ exerted animal studies. TY and NP contributed unpublished reagents and materials. J-CL, SL, G-JS, and BZ analyzed data. Y-LL, J-CL, and BZ wrote the manuscript.

## Conflict of Interest Statement

The authors declare that the research was conducted in the absence of any commercial or financial relationships that could be construed as a potential conflict of interest.

## Footnotes

1 http://www.broadinstitute.org/gsea/msigdb/index.jsp

2 https://www.oncomine.org/

## Supplementary Material

**FIGURE S1 |** Knocking down *NUDT21* suppresses glioblastoma cell proliferation. **(A)** *NUDT21* knockdown was accomplished using shRNA in glioma cells (mean ± SD, *n* = 3, ^∗∗^*p* < 0.01). **(B)** MTT assay showed that, compared with the cell proliferation in the control cells, *NUDT21* knockdown significantly decreased cell proliferation in U251 cells. The OD values were measured at indicated days (mean ± SD, *n* = 3, ns, significant; ^∗∗∗^*p* < 0.001, ^∗∗∗∗^*p* < 0.0001). **(C)** Celigo assay was performed to detect the cell viability over 5 days after transfection. The colony numbers of U251 cells transfected with shCtrl were evidently higher than those transfected with shNUDT21 (mean ± SD, *n* = 3, ns, significant; ^∗∗∗∗^*p* < 0.0001). **(D)** To further determine the physiological role of *NUDT21* in cell growth, U251 cells were transfected with shNUDT21. After 48 h, the apoptotic rates of cells were detected by flow cytometry (mean ± SD, *n* = 3, ^∗∗^*p* < 0.01). **(E)** Flow cytometry analysis was performed to further examine the effect of *NUDT21* on the proliferation and altered cell cycle progression of GBM cells. The bar chart represented the percentage of cells in G0/G1, S or G2/M phase (mean ± SD, *n* = 3, ns, significant; ^∗^*p* < 0.05, ^∗∗∗^*p* < 0.001 versus shCtrl group at indicated phase).

**FIGURE S2 |***NUDT21* knockdown inhibits xenograft tumorigenicity *in vivo* in nude mice. U251 cells were injected into the flanks of the nude mice. **(A)** Tumor volumes were measured on the indicated days (mean ± SD, *n* = 10, ns, significant; ^∗∗∗∗^*p* < 0.0001 versus shCtrl group at indicated days). **(B)** Tumor weights were measured 16 days after injection (mean ± SD, *n* = 10, ^∗∗∗^*p* < 0.001). **(C)** RT-qPCR analysis of *NUDT21* expression in tumor tissues transfected with shNUDT21 or shCtrl. The relative levels of *NUDT21* mRNA were normalized to GAPDH and compared with the expression in tissues transfected with shCtrl (mean ± SD, *n* = 3, ^∗∗∗^*p* < 0.001). **(D)** Immunohistochemistry (IHC) analysis of *NUDT21* expression in tumors developed from shNUDT21-transfected U251 cells and tumors formed by shCtrl-transfected U251 cells. Semi-quantitative analysis of the stained sections was performed using light microscopy to calculate the percentage of positive PCNA staining (mean ± SD, *n* = 3, ^∗∗^*p* < 0.001).

**TABLE S1 |** Microarray analysis comparing the gene expression of U87MG shCtrl versus U87MG shNUDT21 cells.

**TABLE S2 |** General feature of patients.

## References

[B1] Angileri F. F. Aguennouz M. Conti A. La Torre D. Cardali S. Crupi R. (2008). Nuclear factor-κB activation and differential expression of survivin and Bcl-2 in human grade 2–4 astrocytomas. *Cancer* 112 2258–2266. 10.1002/cncr.23407 18327814

[B2] Bao S. Wu Q. McLendon R. E. Hao Y. Shi Q. Hjelmeland A. B. (2006). Glioma stem cells promote radioresistance by preferential activation of the DNA damage response. *Nature* 444 756–760. 10.1038/nature05236 17051156

[B3] Basseres D. S. Baldwin A. S. (2006). Nuclear factor-κB and inhibitor of κB kinase pathways in oncogenic initiation and progression. *Oncogene* 25 6817–6830. 10.1038/sj.onc.1209942 17072330

[B4] Baud V. Karin M. (2009). Is NF-kappaB a good target for cancer therapy? Hopes and pitfalls. *Nat. Rev. Drug Discov.* 8 33–40. 10.1038/nrd2781 19116625 PMC2729321

[B5] Beier D. Schulz J. B. Beier C. P. (2011). Chemoresistance of glioblastoma cancer stem cells–much more complex than expected. *Mol. Cancer* 10:128. 10.1186/1476-4598-10-128 21988793 PMC3207925

[B6] Bhat K. P. Balasubramaniyan V. Vaillant B. Ezhilarasan R. Hummelink K. Hollingsworth F. (2013). Mesenchymal differentiation mediated by NF-κB promotes radiation resistance in glioblastoma. *Cancer Cell* 24 331–346. 10.1016/j.ccr.2013.08.001 23993863 PMC3817560

[B7] Bhat K. P. Salazar K. L. Balasubramaniyan V. Wani K. Heathcock L. Hollingsworth F. (2011). The transcriptional coactivator TAZ regulates mesenchymal differentiation in malignant glioma. *Genes Dev.* 25 2594–2609. 10.1101/gad.176800.111 22190458 PMC3248681

[B8] Capper D. Gaiser T. Hartmann C. Habel A. Mueller W. Herold-Mende C. (2009). Stem-cell-like glioma cells are resistant to TRAIL/Apo2L and exhibit down-regulation of caspase-8 by promoter methylation. *Acta Neuropathol.* 117 445–456. 10.1007/s00401-009-0494-3 19214542

[B9] Carro M. S. Lim W. K. Alvarez M. J. Bollo R. J. Zhao X. Snyder E. Y. (2010). The transcriptional network for mesenchymal transformation of brain tumors. *Nature* 463 318–325. 10.1038/nature08712 20032975 PMC4011561

[B10] Colman H. Zhang L. Sulman E. P. McDonald J. M. Shooshtari N. L. Rivera A. (2010). A multigene predictor of outcome in glioblastoma. *Neuro Oncol.* 12 49–57. 10.1093/neuonc/nop007 20150367 PMC2940562

[B11] Dettwiler S. Aringhieri C. Cardinale S. Keller W. Barabino S. M. (2004). Distinct sequence motifs within the 68-kDa subunit of cleavage factor Im mediate RNA binding, protein-protein interactions, and subcellular localization. *J. Biol. Chem.* 279 35788–35797. 10.1074/jbc.M403927200 15169763

[B12] Elkon R. Ugalde A. P. Agami R. (2013). Alternative cleavage and polyadenylation: extent, regulation and function. *Nat. Rev. Genet.* 14 496–506. 10.1038/nrg3482 23774734

[B13] Etminan N. Peters C. Ficnar J. Anlasik S. Bünemann E. Slotty P. J. (2011a). Modulation of migratory activity and invasiveness of human glioma spheroids following 5-aminolevulinic acid-based photodynamic treatment. Laboratory investigation. *J. Neurosurg.* 115 281–288. 10.3171/2011.3.JNS10434 21513432

[B14] Etminan N. Peters C. Lakbir D. Bünemann E. Börger V. Sabel M. C. (2011b). Heat-shock protein 70-dependent dendritic cell activation by 5-aminolevulinic acid-mediated photodynamic treatment of human glioblastoma spheroids *in vitro*. *Br. J. Cancer* 105 961–969. 10.1038/bjc.2011.327 21863026 PMC3185943

[B15] Eto A. Muta T. Yamazaki S. Takeshige K. (2003). Essential roles for NF-kappa B and a Toll/IL-1 receptor domain-specific signal(s) in the induction of I kappa B-zeta. *Biochem. Biophys. Res. Commun.* 301 495–501. 10.1016/S0006-291X(02)03082-6 12565889

[B16] Furnari F. B. Fenton T. Bachoo R. M. Mukasa A. Stommel J. M. Stegh A. (2007). Malignant astrocytic glioma: genetics, biology, and paths to treatment. *Genes Dev.* 21 2683–2710. 10.1101/gad.1596707 17974913

[B17] Gennarino V. A. Alcott C. E. Chen C. A. Chaudhury A. Gillentine M. A. Rosenfeld J. A. (2015). NUDT21-spanning CNVs lead to neuropsychiatric disease and altered MeCP2 abundance via alternative polyadenylation. *eLife* 4:e10782. 10.7554/eLife.10782 26312503 PMC4586391

[B18] Greten F. R. Arkan M. C. Bollrath J. Hsu L. C. Goode J. Miething C. (2007). NF-kappaB is a negative regulator of IL-1beta secretion as revealed by genetic and pharmacological inhibition of IKKbeta. *Cell* 130 918–931. 10.1016/j.cell.2007.07.009 17803913 PMC2134986

[B19] Halliday J. Helmy K. Pattwell S. S. Pitter K. L. LaPlant Q. Ozawa T. (2014). In vivo radiation response of proneural glioma characterized by protective p53 transcriptional program and proneural-mesenchymal shift. *Proc. Natl. Acad. Sci. U.S.A.* 111 5248–5253. 10.1073/pnas.1321014111 24706837 PMC3986190

[B20] Hemmati H. D. Nakano I. Lazareff J. A. Masterman-Smith M. Geschwind D. H. Bronner-Fraser M. (2003). Cancerous stem cells can arise from pediatric brain tumors. *Proc. Natl. Acad. Sci. U.S.A.* 100 15178–15183. 10.1073/pnas.2036535100 14645703 PMC299944

[B21] Hoffmann A. Baltimore D. (2006). Circuitry of nuclear factor κB signaling. *Immunol. Rev.* 210 171–186. 10.1111/j.0105-2896.2006.00375.x 16623771

[B22] Karin M. (2006). NF-κB and cancer: mechanisms and targets. *Mol. Carcinog.* 45 355–361. 10.1002/mc.20217 16673382

[B23] Kim S. H. Ezhilarasan R. Phillips E. Gallego-Perez D. Sparks A. Taylor D. (2016). Serine/threonine kinase MLK4 determines mesenchymal identity in glioma stem cells in an NF-κB-dependent manner. *Cancer Cell* 29 201–213. 10.1016/j.ccell.2016.01.005 26859459 PMC4837946

[B24] Korkolopoulou P. Levidou G. Saetta A. A. El-Habr E. Eftichiadis C. Demenagas P. (2008). Expression of nuclear factor-kappaB in human astrocytomas: relation to pI kappa Ba, vascular endothelial growth factor, Cox-2, microvascular characteristics, and survival. *Hum. Pathol.* 39 1143–1152. 10.1016/j.humpath.2008.01.020 18495209

[B25] Mao P. Joshi K. Li J. Kim S. H. Li P. Santana-Santos L. (2013). Mesenchymal glioma stem cells are maintained by activated glycolytic metabolism involving aldehyde dehydrogenase 1A3. *Proc. Natl. Acad. Sci. U.S.A.* 110 8644–8649. 10.1073/pnas.1221478110 23650391 PMC3666732

[B26] Masamha C. P. Xia Z. Yang J. Albrecht T. R. Li M. Shyu A. B. (2014). FIm25 links alternative polyadenylation to glioblastoma tumour suppression. *Nature* 510 412–416. 24814343 10.1038/nature13261 PMC4128630

[B27] Naganuma T. Nakagawa S. Tanigawa A. Sasaki Y. F. Goshima N. Hirose T. (2012). Alternative 3′-end processing of long noncoding RNA initiates construction of nuclear paraspeckles. *EMBO J.* 31 4020–4034. 10.1038/emboj.2012.251 22960638 PMC3474925

[B28] Nozell S. Laver T. Moseley D. Nowoslawski L. De Vos M. Atkinson G. P. (2008). The ING4 tumor suppressor attenuates NF-κB activity at the promoters of target genes. *Mol. Cell. Biol.* 28 6632–6645. 10.1128/MCB.00697-08 18779315 PMC2573235

[B29] Nurwidya F. Takahashi F. Murakami A. Takahashi K. (2012). Epithelial mesenchymal transition in drug resistance and metastasis of lung cancer. *Cancer Res. Treat.* 44 151–156. 10.4143/crt.2012.44.3.151 23091440 PMC3467417

[B30] Pelloski C. E. Mahajan A. Maor M. Chang E. L. Woo S. Gilbert M. (2005). YKL-40 expression is associated with poorer response to radiation and shorter overall survival in glioblastoma. *Clin. Cancer Res.* 11 3326–3334. 10.1158/1078-0432.CCR-04-1765 15867231

[B31] Phillips H. S. Kharbanda S. Chen R. Forrest W. F. Soriano R. H. Wu T. D. (2006). Molecular subclasses of high-grade glioma predict prognosis, delineate a pattern of disease progression, and resemble stages in neurogenesis. *Cancer Cell* 9 157–173. 10.1016/j.ccr.2006.02.019 16530701

[B32] Robe P. A. Bentires-Alj M. Bonif M. Rogister B. Deprez M. Haddada H. (2004). *In vitro* and *in vivo* activity of the nuclear factor-κB inhibitor sulfasalazine in human glioblastomas. *Clin. Cancer Res*. 10 5595–5603. 10.1158/1078-0432.CCR-03-0392 15328202

[B33] Ruegsegger U. Beyer K. Keller W. (1996). Purification and characterization of human cleavage factor Im involved in the 3′ end processing of messenger RNA precursors. *J. Biol. Chem.* 271 6107–6113. 10.1074/jbc.271.11.6107 8626397

[B34] Sanchez-Tillo E. Liu Y. de Barrios O. Siles L. Fanlo L. Cuatrecasas M. (2012). EMT-activating transcription factors in cancer: beyond EMT and tumor invasiveness. *Cell. Mol. Life Sci.* 69 3429–3456. 10.1007/s00018-012-1122-2 22945800 PMC11115078

[B35] Shirkoohi R. (2013). Epithelial mesenchymal transition from a natural gestational orchestration to a bizarre cancer disturbance. *Cancer Sci.* 104 28–35. 10.1111/cas.12074 23181983 PMC7657227

[B36] Singh S. K. Clarke I. D. Terasaki M. Bonn V. E. Hawkins C. Squire J. (2003). Identification of a cancer stem cell in human brain tumors. *Cancer Res.* 63 5821–5828. 14522905

[B37] Singh S. K. Hawkins C. Clarke I. D. Squire J. A. Bayani J. Hide T. (2004). Identification of human brain tumour initiating cells. *Nature* 432 396–401. 10.1038/nature03128 15549107

[B38] Sowers J. L. Johnson K. M. Conrad C. Patterson J. T. Sowers L. C. (2014). The role of inflammation in brain cancer. *Adv. Exp. Med. Biol.* 816 75–105. 10.1007/978-3-0348-0837-8_4 24818720

[B39] Stewart L. A. (2002). Chemotherapy in adult high-grade glioma: a systematic review and meta-analysis of individual patient data from 12 randomised trials. *Lancet* 359 1011–1018. 10.1016/S0140-6736(02)08091-1 11937180

[B40] Subramanian A. Tamayo P. Mootha V. K. Mukherjee S. Ebert B. L. Gillette M. A. (2005). Gene set enrichment analysis: a knowledge-based approach for interpreting genome-wide expression profiles. *Proc. Natl. Acad. Sci. U.S.A.* 102 15545–15550. 10.1073/pnas.0506580102 16199517 PMC1239896

[B41] Totzke G. Essmann F. Pohlmann S. Lindenblatt C. Janicke R. U. Schulze-Osthoff K. (2006). A novel member of the IkappaB family, human IkappaB-zeta, inhibits transactivation of p65 and its DNA binding. *J. Biol. Chem.* 281 12645–12654. 10.1074/jbc.M511956200 16513645

[B42] Venkataraman K. Brown K. M. Gilmartin G. M. (2005). Analysis of a noncanonical poly(A) site reveals a tripartite mechanism for vertebrate poly(A) site recognition. *Genes Dev.* 19 1315–1327. 10.1101/gad.1298605 15937220 PMC1142555

[B43] Vescovi A. L. Galli R. Reynolds B. A. (2006). Brain tumour stem cells. *Nat. Rev. Cancer* 6 425–436. 10.1038/nrc1889 16723989

[B44] Wang H. Zhang W. Huang H. J. Liao W. S. Fuller G. N. (2004). Analysis of the activation status of Akt, NFkappaB, and Stat3 in human diffuse gliomas. *Lab. Invest.* 84 941–951. 10.1038/labinvest.3700123 15184909

[B45] Yamazaki S. Muta T. Takeshige K. (2001). A novel IkappaB protein, IkappaB-zeta, induced by proinflammatory stimuli, negatively regulates nuclear factor-kappaB in the nuclei. *J. Biol. Chem.* 276 27657–27662. 10.1074/jbc.M103426200 11356851

[B46] Zhong J. Paul A. Kellie S. J. O’Neill G. M. (2010). Mesenchymal migration as a therapeutic target in glioblastoma. *J. Oncol.* 2010:430142. 10.1155/2010/430142 20652056 PMC2905941

