# Supplementary figures and images for: Silencing NUDT21 Attenuates the Mesenchymal Identity of Glioblastoma Cells via the NF-κB Pathway
Source: Front Mol Neurosci. 2017 Dec 19;10:420. doi: 10.3389/fnmol.2017.00420 (PMC5742174; doi:10.3389/fnmol.2017.00420)

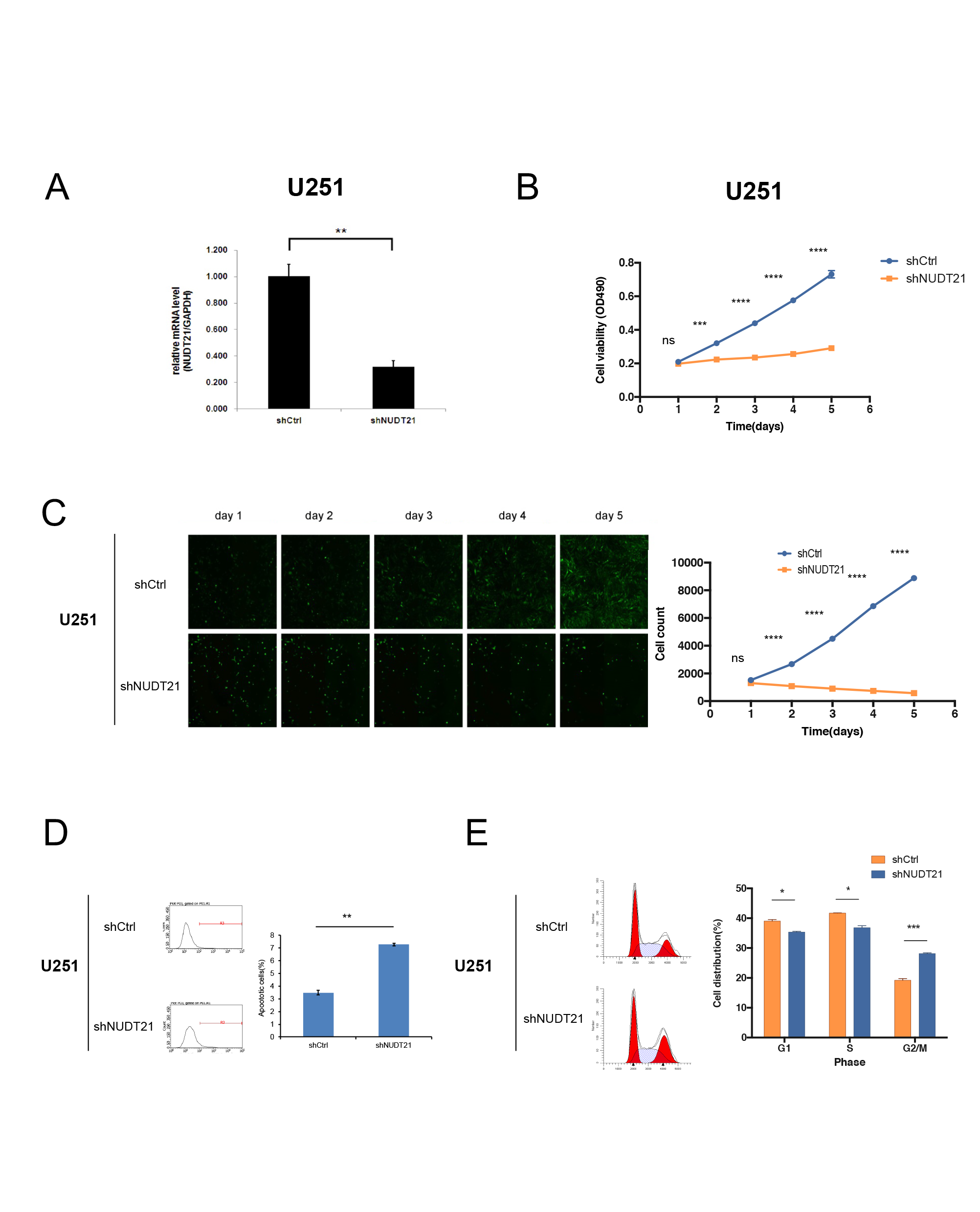

Supplement: Supplementary file 1 [file Image_1.tif]

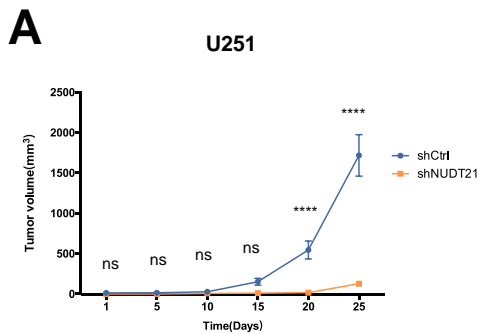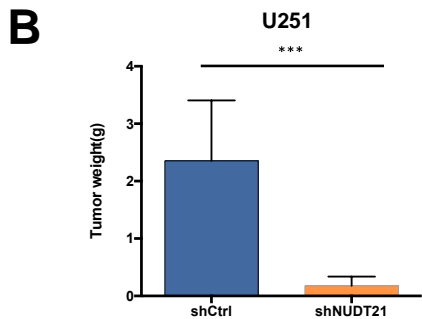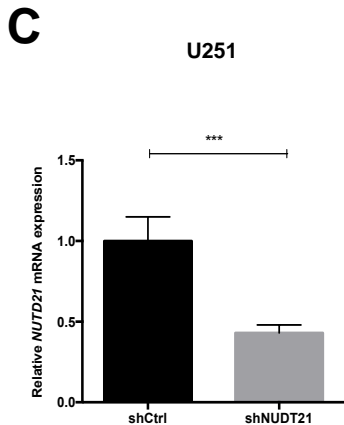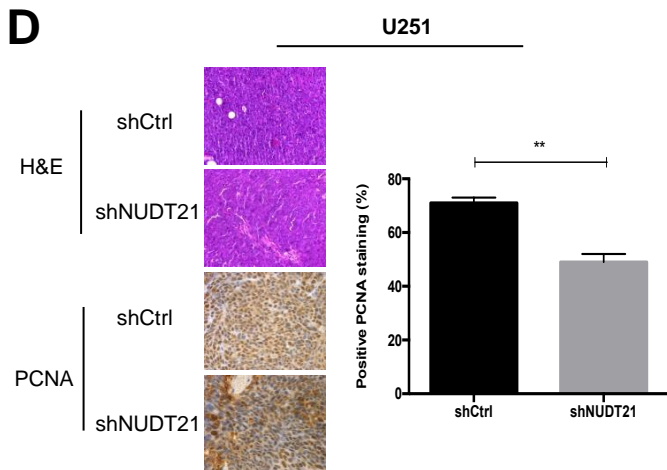

Supplement: Supplementary file 2 [file Image_2.PDF]
