# Supplementary material for: Silencing NUDT21 Attenuates the Mesenchymal Identity of Glioblastoma Cells via the NF-κB Pathway
Source: Front Mol Neurosci. 2017 Dec 19;10:420. doi: 10.3389/fnmol.2017.00420 (PMC5742174; doi:10.3389/fnmol.2017.00420)
Supplement: Supplementary file 3 [file Table_1.PDF]

Supplementary Table S1. Microarray analysis comparing the gene expression of U87MG shCtrl versus U87MG shNUDT21 cells.

Probe	KD			NC			P-value		Fold change				Annotation					
probeSet	1081-3_(PrimeView).CEL	1081-2_(PrimeView).CEL	1081-1_(PrimeView).CEL	1080-3_(PrimeView).CEL	1080-2_(PrimeView).CEL	1080-1_(PrimeView).CEL	Corrected p-value	P-value	Fold change	Log Fold change	Absolute Fold change	Regulation	Gene Symbol	Entrez Gene	Gene Ontology Biological Process	Gene Ontology Cellular Component	Gene Ontology Molecular Function	Alignments
11730691_s_at	-0.34622526	-0.28440952	-0.5211668	0.38773918	0.39857578	0.28440952	0.001643622	0.00074487	-1.671151	-0.74084204	1.6711509	down	ALS2	57679	0001662 // behavioral fear	0001726 // ruffle // inferred	0005085 // guanyl-	chr2:202624179-
11715497_a_at	-0.25009346	-0.38500977	-0.39699078	0.28715038	0.30519772	0.25009346	0.001105608	0.00023237	-1.542045	-0.62484515	1.5420454	down	OXA1L	5018	0006461 // protein complex	0005739 // mitochondrion //	0005515 // protein binding //	chr14:23235730-23240970
11744468_at	-0.3860736	-0.42154598	-0.63731575	0.4524846	0.42838955	0.3860736	0.001295644	0.00036465	-1.871197	-0.90396106	1.8711965	down	SYNCRIP	10492	0000398 // nuclear mRNA	0005634 // nucleus //	0000166 // nucleotide	chr6:86317320-86352709 (-
11733815_at	-0.4549036	-0.2495656	-0.30994558	0.39776945	0.30902624	0.2495656	0.001821452	0.00091792	-1.576719	-0.6569253	1.5767187	down	RPL22L1	200916	0006412 // translation //	0005622 // intracellular //	0003735 // structural	chr3:170582666-
11764283_s_at	-0.21599579	-0.327878	-0.4552536	0.21599531	0.2668066	0.3062787	0.002244884	0.00128261	-1.511593	-0.59606934	1.5115926	down	PNISR	25957		0005634 // nucleus //		chr6:99846538-99847045 (-
11739456_at	0.41606522	0.37517452	0.31555748	-0.31555796	-0.3292575	-0.3860097	0.000660041	3.9664E-05	1.6386875	0.71254075	1.6386875	up	SGMS1	259230	0006629 // lipid metabolic	0000138 // Golgi trans	0016301 // kinase activity //	chr10:52065359-52383737
11726244_a_at	0.49816608	0.53118515	0.4986925	-0.5746937	-0.49816608	-0.5881376	0.000418755	3.8226E-06	2.0892916	1.0630138	2.0892916	up	RORA	6095	0006351 // transcription,	0005634 // nucleus //	0003677 // DNA binding //	chr15:60780482-60919729
11725706_s_at	-0.27396345	-0.22588778	-0.5574093	0.35132647	0.36662197	0.22588778	0.005203444	0.00407207	-1.587803	-0.66703224	1.5878034	down	AMOTL1	154810	0003365 // establishment of	0005737 // cytoplasm //	0005515 // protein binding //	chr11:94501471-94609917
11743337_at	-0.24191427	-0.36270285	-0.4552164	0.2965684	0.24191427	0.36147928	0.001664025	0.00076538	-1.572724	-0.6532651	1.5727236	down	PPAPDC2	403313	0008152 // metabolic	0016020 // membrane //	0003824 // catalytic activity	chr9:4662288-4665268 (+)
11723957_x_at	-0.22684717	-0.3738742	-0.6750455	0.278821	0.22684717	0.28709698	0.007822415	0.00663118	-1.612737	-0.6895107	1.6127365	down	LSM5	23658	0000288 // nuclear-	0005634 // nucleus //	0003723 // RNA binding //	chr7:32525083-32530028 (-
11733059_a_at	0.33220243	0.41532564	0.29538918	-0.50167656	-0.29538918	-0.4172964	0.001334832	0.00041521	1.6846238	0.7524265	1.6846238	up	PTBP3	9991	0006397 // mRNA	0005634 // nucleus //	0000166 // nucleotide	chr9:114980718-
11757504_a_at	-0.4106865	-0.35271692	-0.46214437	0.3765087	0.40793705	0.35271692	0.000581859	2.4226E-05	-1.726165	-0.7875701	1.7261647	down	DDAH1	23576	0000052 // citrulline	0005737 // cytoplasm //	0003824 // catalytic activity	chr1:85784167-85784590 (-
11719312_a_at	0.49019003	0.35321093	0.25002527	-0.25002527	-0.32725286	-0.3453579	0.001775921	0.00087826	1.5933031	0.67202073	1.5933031	up	MAP2K5	5607	0000122 // negative	0005634 // nucleus //	0000166 // nucleotide	chr15:67834943-68099452
11732491_s_at	0.4133129	0.38947058	0.3458786	-0.34587908	-0.3680668	-0.59327984	0.001434982	0.00055131	1.7637295	0.81862926	1.7637295	up	BRAF	673	0000165 // MAPK cascade	0005624 // membrane	0000166 // nucleotide	chr7:140433306-
11728787_a_at	-0.36708927	-0.26868486	-0.27604437	0.35601377	0.40170097	0.26868486	0.001094578	0.00021083	-1.564902	-0.6460727	1.5649024	down	PTPRR	5801	0001701 // in utero	0005634 // nucleus //	0004721 // phosphoprotein	chr12:71031853-71314623
11743484_at	-0.40982723	-0.26867056	-0.38015842	0.26867056	0.29232883	0.2694583	0.000954117	0.00013437	-1.547248	-0.6297046	1.5472481	down	NAA25	80018		0005737 // cytoplasm //		chr12:112464492-
11729450_at	-0.3422823	-0.2983694	-0.30630207	0.2983694	0.4012499	0.36328316	0.000637897	3.4488E-05	-1.59102	-0.6699521	1.5910201	down	PCLO	27445	0006810 // transport //	0005856 // cytoskeleton //	0005215 // transporter	chr7:82449797-82792197 (-
11729626_a_at	0.33308315	0.2899227	0.32997227	-0.37000847	-0.2899227	-0.36117554	0.000581859	2.2073E-05	1.5779246	0.65802824	1.5779246	up	DCAF7	10238	0007275 // multicellular	0005634 // nucleus //	0005515 // protein binding //	chr17:61627800-61671630
11757723_a_at	0.35699034	0.39301634	0.29259443	-0.29259443	-0.32329893	-0.3293662	0.000611709	3.0189E-05	1.5829549	0.6626202	1.5829549	up	CD163L1	283316		0005576 // extracellular	0004872 // receptor activity	chr12:7507555-7520765 (-)
11745377_x_at	-0.3207655	-0.24950218	-0.5801878	0.24991512	0.24950218	0.2969265	0.004181807	0.00309565	-1.568008	-0.6489331	1.5680082	down	PTRF	284119	0006351 // transcription,	0005634 // nucleus //	0003723 // RNA binding //	chr17:40555653-40575189
11763274_a_at	-0.29071093	-0.23576069	-0.6631913	0.30507374	0.23576117	0.26242828	0.009301878	0.00808471	-1.584809	-0.6643087	1.5848087	down	SIPA1L1	26037	0031532 // actin	0005622 // intracellular //	0005096 // GTPase	chr14:72052997-72207926
11737490_a_at	-0.23181725	-0.24752569	-0.21452713	0.21452713	0.42641258	0.4362259	0.002224211	0.0012669	-1.505607	-0.5903452	1.505607	down	IL36B	27177	0006955 // immune	0005576 // extracellular	0005125 // cytokine activity	chr2:113785009-
11726855_at	-0.45626736	-0.49085903	-0.60730886	0.7252617	0.5980611	0.45626688	0.001115206	0.00024854	-2.160465	-1.1113417	2.1604648	down	ADAMTS5	11096	0006508 // proteolysis //	0005576 // extracellular	0004222 //	chr21:28290240-28339439
11739834_s_at	-0.39600372	-0.22538757	-0.34540558	0.33333778	0.25536537	0.22538757	0.00147514	0.00058407	-1.509038	-0.59362924	1.5090381	down	MOB4	100529241 ///	0006810 // transport //	0005624 // membrane	0005515 // protein binding //	chr2:198380770-
11725861_a_at	0.4987917	0.5304327	0.50477695	-0.5050645	-0.62599945	-0.49879122	0.000566185	1.5682E-05	2.0771694	1.0546188	2.0771694	up	MYBL1	4603	0006351 // transcription,	0005634 // nucleus //	0003677 // DNA binding //	chr8:67474410-67525484 (-
11728361_a_at	-0.25634098	-0.26385212	-0.3814969	0.37125492	0.25634098	0.3398924	0.001193643	0.0003015	-1.540138	-0.6230594	1.5401378	down	CHCHD7	79145		0005739 // mitochondrion //	0004129 // cytochrome-c	chr8:57124314-57131357
11716184_s_at	0.34896946	0.24854851	0.25573158	-0.38008022	-0.32917166	-0.24854898	0.001152694	0.00027249	1.5195915	0.6036835	1.5195915	up	ALCAM	214	0007155 // cell adhesion //	0009897 // external side of	0005102 // receptor binding	chr3:105085712-
11751738_a_at	-0.25073814	-0.5234823	-0.4423046	0.25073814	0.32579184	0.3060255	0.002063705	0.00113079	-1.62416	-0.69969356	1.6241598	down	PPP1R3E	90673	0005975 // carbohydrate	0042587 // glycogen	0004721 // phosphoprotein	chr14:23767460-23770734
11744716_a_at	-0.357718	-0.32402945	-0.48725605	0.5483608	0.32402992	0.60569954	0.001785929	0.00088642	-1.843394	-0.88236463	1.8433942	down	PFN2	5217	0007010 // cytoskeleton	0005737 // cytoplasm //	0003779 // actin binding //	chr3:149682690-
11739508_a_at	-0.31680584	-0.37029266	-0.46524334	0.4417801	0.41004848	0.31680584	0.001044088	0.00017488	-1.7096	-0.77365875	1.7096	down	NAA30	122830		0005737 // cytoplasm //	0004596 // peptide alpha-N-	chr14:57857270-57879699
11727815_at	0.4020319	0.36596584	0.25730133	-0.3296218	-0.25730133	-0.33278847	0.001086023	0.00020403	1.5673603	0.6483369	1.5673603	up	UBASH3B	84959	0035335 // peptidyl-tyrosine	0005634 // nucleus //	0004721 // phosphoprotein	chr11:122525899-
11718089_at	0.32216358	0.4430542	0.2541256	-0.2541256	-0.3107009	-0.27995062	0.001336215	0.00041917	1.538339	0.62137353	1.538339	up	TNKS	8658	0000209 // protein	0000139 // Golgi membrane	0003950 // NAD+ ADP-	chr8:9412656-9639856 (+)
11746242_a_at	0.39348125	0.35984898	0.32122135	-0.45913982	-0.36971188	-0.32122135	0.000847916	8.2735E-05	1.6719613	0.7415415	1.6719613	up	ARNTL2	56938	0006351 // transcription,	0005634 // nucleus //	0000982 // RNA	chr12:27485987-27574195
11755059_s_at	0.28242588	0.382411	0.27293205	-0.27293205	-0.3780775	-0.40398216	0.001107964	0.00023692	1.5847481	0.6642536	1.5847481	up	TCF3	6929	0000080 // G1 phase of	0000788 // nuclear	0003677 // DNA binding //	chr19:1611055-1619149 (-)
11719784_x_at	-0.30074406	-0.28510952	-0.3332796	0.33760452	0.28510952	0.373641	0.000587439	2.6449E-05	-1.556706	-0.6384961	1.5567056	down	RPS23	6228	0000184 // nuclear-	0005622 // intracellular //	0003735 // structural	chr5:81569560-81574396 (-
11722108_at	-0.28522444	-0.26950693	-0.37107325	0.31126451	0.2759738	0.26950693	0.000767869	6.3881E-05	-1.509618	-0.59418327	1.5096177	down	PCNX	22990		0016020 // membrane //		chr14:71374121-71582098
11746441_a_at	0.39951944	0.35694265	0.34930468	-0.34930468	-0.5201893	-0.41230583	0.000894235	0.0001086	1.7361066	0.7958555	1.7361066	up	TGFBR1	7046	0000186 // activation of	0005886 // plasma	0000166 // nucleotide	chr9:101891135-
11733093_a_at	-0.3109746	-0.3666649	-0.3437581	0.53641415	0.31097507	0.3484707	0.001406785	0.0004967	-1.669118	-0.73908585	1.6691179	down	FOXRED2	80020	0030433 // ER-associated	0005783 // endoplasmic	0005515 // protein binding //	chr22:36883233-36903090
11753214_a_at	0.3123765	0.29535055	0.27182198	-0.2718215	-0.34439707	-0.28290606	0.000580195	2.0125E-05	1.5082663	0.5928912	1.5082663	up	PIBF1	10464		0005813 // centrosome //		chr13:73356362-73590118
11718511_s_at	-0.44443417	-0.454751	-0.26730537	0.36208534	0.30783844	0.26730537	0.001381386	0.00046403	-1.625902	-0.70123994	1.6259016	down	UBE2L3	7332	0000209 // protein	0000151 // ubiquitin ligase	0000166 // nucleotide	chr22:21921956-21978323
11745016_a_at	-0.32760334	-0.39367294	-0.37371826	0.38434124	0.32760334	0.38009453	0.000555711	1.0784E-05	-1.657503	-0.72901124	1.6575028	down	C1orf43	25912	0055114 // oxidation-	0016020 // membrane //	0016491 // oxidoreductase	chr1:154183936-
11723091_s_at	-0.45346165	-0.25886726	-0.30640507	0.3419838	0.26747227	0.25886726	0.001498424	0.00060976	-1.546513	-0.62901914	1.5465132	down	FNIP2	57600	0001932 // regulation of	0005634 // nucleus //	0005515 // protein binding //	chr4:159690165-
11736986_x_at	0.3708005	0.42071533	0.20399523	-0.2039957	-0.29075956	-0.27474785	0.002077202	0.0011417	1.5035138	0.5883381	1.5035138	up	NUBPL	80224	0032981 // mitochondrial	0005739 // mitochondrion //	0000166 // nucleotide	chr14:32030590-32330368
11741572_a_at	0.5894575	0.36007118	0.40844917	-0.36007118	-0.47079468	-0.5295429	0.001375919	0.00045243	1.8740103	0.9061289	1.8740103	up	RGS4	5999	0000188 // inactivation of	0005624 // membrane	0005096 // GTPase	chr1:163041694-
11736559_a_at	-0.3166957	-0.3840251	-0.3979206	0.31669617	0.37113714	0.4926815	0.001075945	0.00019284	-1.693161	-0.7597188	1.6931605	down	MAP2	4133	0001578 // microtubule	0005622 // intracellular //	0002162 // dystroglycan	chr2:210444144-
11731782_s_at	0.46394634	0.40568924	0.44163418	-0.4655943	-0.4268551	-0.40568924	0.000418755	3.6994E-06	1.8274131	0.86980283	1.8274131	up	CT45A2	441519 ///				chrX:134928583-
11723821_a_at	0.4098215	0.45317173	0.45210648	-0.43995667	-0.4098215	-0.41073513	0.000309231	1.0027E-06	1.8131995	0.8585377	1.8131995	up	SMURF2	64750	0006464 // cellular protein	0000151 // ubiquitin ligase	0004842 // ubiquitin-protein	chr17:62540734-62658460
11731782_s_at	0.46394634	0.40568924	0.44163418	-0.4655943	-0.4268551	-0.40568924	0.000418755	3.6994E-06	1.8274131	0.86980283	1.8274131	up	CT45A1	441519 ///				chrX:134928583-
11731782_s_at	0.46394634	0.40568924	0.44163418	-0.4655943	-0.4268551	-0.40568924	0.000418755	3.6994E-06	1.8274131	0.86980283	1.8274131	up	CT45A4	441519 ///				chrX:134928583-
11731782_s_at	0.46394634	0.40568924	0.44163418	-0.4655943	-0.4268551	-0.40568924	0.000418755	3.6994E-06	1.8274131	0.86980283	1.8274131	up	CT45A3	441519 ///				chrX:134928583-
11734212_at	0.51050854	0.4542694	0.4383955	-0.5182934	-0.4966154	-0.4383955	0.000534553	7.9512E-06	1.9347662	0.9521592	1.9347662	up	CT45A6	441521 ///				chrX:134887165-
11743841_at	0.35349083	0.27148104	0.39692545	-0.27148104	-0.3980999	-0.7113595	0.005272855	0.00413531	1.742243	0.8009459	1.742243	up	PLCXD2	257068	0016042 // lipid catabolic	0005622 // intracellular //	0004629 // phospholipase C	chr3:111393506-
11734212_at	0.51050854	0.4542694	0.4383955	-0.5182934	-0.4966154	-0.4383955	0.000534553	7.9512E-06	1.9347662	0.9521592	1.9347662	up	CT45A5	441521 ///				chrX:134887165-
11725211_a_at	-0.23209715	-0.30201244	-0.36739588	0.40645504	0.3194523	0.23209715	0.001512136	0.00062515	-1.536701	-0.6198367	1.5367012	down	PLCXD1	55344	0006629 // lipid metabolic	0005622 // intracellular //	0004629 // phospholipase C	chrX:198144-220020 (+) //
11758600_s_at	-0.22415876	-0.3416958	-0.41794634	0.34328127	0.25940037	0.22415876	0.001718428	0.00081802	-1.519448	-0.6035471	1.5194478	down	SRSF1	6426	0000395 // nuclear mRNA	0005634 // nucleus //	0000166 // nucleotide	chr17:56078281-56078671
11736685_a_at	0.3263216	0.450799	0.3173523	-0.4210167	-0.31735182	-0.4671893	0.001108536	0.00024247	1.7013464	0.7666769	1.7013464	up	FGF5	2250	0007267 // cell-cell signaling	0005576 // extracellular	0005104 // fibroblast growth	chr4:81187696-81212170
11754547_a_at	-0.3549552	-0.34380388	-0.37029982	0.34380388	0.34922123	0.3695836	0.000290487	3.4167E-07	-1.636434	-0.71055585	1.6364344	down	ENOX2	10495	0006810 // transport //	0005576 // extracellular	0000166 // nucleotide	chrX:129757875-
11717599_s_at	-0.3987999	-0.19458294	-0.43470335	0.27214813	0.19458294	0.26352167	0.002747926	0.00173663	-1.501197	-0.586113	1.5011966	down	SLC20A2	6575	0006810 // transport //	0005624 // membrane	0004872 // receptor activity	chr8:42273996-42330175 (-
11748094_a_at	0.23930454	0.3373394	0.33830833	-0.23930454	-0.30361652	-0.3149929	0.000937307	0.00012732	1.5062438	0.5909554	1.5062438	up	FAM3C	10447	0007275 // multicellular	0005576 // extracellular	0005125 // cytokine activity	chrX:23093702-23096457
11748516_a_at	-0.33971834	-0.3897729	-0.43419313	0.33971834	0.40575075	0.4722681	0.000798199	7.2031E-05	-1.733643	-0.79380715	1.7336434	down	NAP1L5	266812	0006334 // nucleosome	0005634 // nucleus //		chr4:89617462-89618952 (-
11757658_a_at	0.31886768	0.42798233	0.39294624	-0.3522129	-0.31886768	-0.35366535	0.000611709	2.9538E-05	1.6489116	0.72151405	1.6489116	up	LZIC	84328	0007417 // central nervous		0008013 // beta-catenin	chr1:9982172-10002807 (-)
11716044_a_at	-0.35409832	-0.38155746	-0.5770893	0.41060638	0.35409832	0.44956303	0.001296612	0.00036688	-1.792953	-0.8423376	1.7929529	down	FAM168B	130074		0005886 // plasma		chr2:131805448-
11733100_at	0.30537271	0.383574	0.24576569	-0.39590454	-0.24576569	-0.48158932	0.00191671	0.00099835	1.6088063	0.68599063	1.6088063	up	USP12	219333	0006464 // cellular protein		0004197 // cysteine-type	chr13:27640292-27746029
11726693_s_at	-0.28536844	-0.26338625	-0.3716445	0.3956461	0.26338625	0.38911247	0.001152694	0.00026813	-1.575906	-0.65618134	1.5759058	down	ANGPT1	284	0001525 // angiogenesis //	0005576 // extracellular	0005102 // receptor binding	chr8:108261718-
11763395_a_at	-0.2812462	-0.28764486	-0.35301352	0.47112608	0.2812462	0.33730507	0.001301856	0.00038463	-1.591655	-0.67052734	1.5916547	down	ZC3HAV1L	92092				chr7:138706244-
11758444_s_at	-0.23870087	-0.42978144	-0.3851595	0.2545643	0.23870087	0.36124325	0.001684569	0.00078528	-1.554069	-0.6360501	1.5540686	down	DHTKD1	55526	0006096 // glycolysis //	0005739 // mitochondrion //	0004591 // oxoglutarate	chr10:12164661-12165152
11725039_at	0.3507738	0.25340557	0.37421227	-0.3104372	-0.26336956	-0.25340557	0.000936118	0.00012476	1.5176804	0.601868	1.5176804	up	FGF2	2247	0000186 // activation of	0005576 // extracellular	0005104 // fibroblast growth	chr4:123747862-
11729915_at	0.27887964	0.539021	0.25386477	-0.25386477	-0.33215594	-0.28196573	0.003382227	0.00234066	1.565457	0.6465839	1.565457	up	ARL5B	221079	0007264 // small GTPase	0005622 // intracellular //	0000166 // nucleotide	chr10:18948263-18967357
11757033_a_at	0.28507423	0.33894634	0.26443386	-0.3716507	-0.26443386	-0.2895441	0.000894235	0.00010371	1.5206566	0.60469437	1.5206566	up	IL13RA2	3598	0007165 // signal	0005615 // extracellular	0004871 // signal	chrX:114239295-
11723123_at	-0.22168732	-0.3135519	-0.50066185	0.2216878	0.29282522	0.3388052	0.003127867	0.00209407	-1.547286	-0.62973976	1.5472859	down	SH3PXD2B	285590	0001501 // skeletal system	0002102 // podosome //	0005515 // protein binding //	chr5:171760502-
11719002_at	-0.3408985	-0.2964325	-0.3153162	0.2964325	0.34349918	0.36906242	0.000556561	1.2434E-05	-1.573395	-0.6538805	1.5733945	down	KDELR3	11015	0006351 // transcription,	0005634 // nucleus //	0000166 // nucleotide	chr22:38864066-38879445
11733944_at	-0.2278099	-0.32333136	-0.735837	0.2278099	0.22830343	0.28962755	0.013731535	0.01255046	-1.599447	-0.677573	1.5994468	down	RAP2B	5912	0006184 // GTP catabolic	0005768 // endosome //	0000166 // nucleotide	chr3:152880014-
11757513_at	0.3475523	0.23214531	0.29300976	-0.3742075	-0.32170916	-0.23214531	0.001284364	0.00035177	1.515986	0.60025644	1.515986	up	NFKBIZ	64332	0006351 // transcription,	0005634 // nucleus //		chr3:101579157-
11725095_at	0.2414217	0.31603336	0.31977892	-0.34795618	-0.29276752	-0.2414217	0.000936118	0.00012551	1.5015576	0.58645976	1.5015576	up	TTC33	23548				chr5:40711699-40756064 (-
11731580_x_at	0.4541011	0.40606928	0.4178276	-0.40606928	-0.66149807	-0.5274296	0.001103597	0.00021869	1.942164	0.95766497	1.942164	up	KCTD20	222658	0051260 // protein		0005216 // ion channel	chr6:36410543-36458918
11759419_at	0.5242486	0.37920523	0.19566774	-0.23282528	-0.19566774	-0.31678724	0.00487323	0.0037628	1.5313463	0.61480063	1.5313463	up	GTF2H3	2967	0000718 // nucleotide-	0000439 // core TFIIH	0003684 // damaged DNA	chr12:124118320-
11730914_a_at	-0.398942	-0.3405075	-0.45109272	0.39081573	0.34154224	0.3405075	0.000616996	3.0624E-05	-1.687011	-0.7544692	1.6870108	down	ERLIN1	10613	0030433 // ER-associated	0005783 // endoplasmic	0005515 // protein binding //	chr10:101909846-
11736006_at	-0.40884876	-0.35802746	-0.5284109	0.39010906	0.35802746	0.37196255	0.000884071	9.6635E-05	-1.747302	-0.8051287	1.7473017	down	MAPK1IP1L	93487				chr14:55518355-55536912
11725654_at	-0.5526483	-0.28143263	-0.33045053	0.37915945	0.28143263	0.30621147	0.002271868	0.00130379	-1.636309	-0.71044505	1.6363088	down	GTF2H5	404672	0006281 // DNA repair //	0005634 // nucleus //	0003677 // DNA binding //	chr6:158589365-
11727293_x_at	-0.2469368	-0.38180304	-0.38990688	0.2872734	0.28051376	0.2469368	0.001103597	0.00021949	-1.527448	-0.61112356	1.5274483	down	ALG10B	144245	0006486 // protein	0005886 // plasma	0016740 // transferase	chr12:38710556-38723793
11733997_a_at	0.3313446	0.3494997	0.3012581	-0.4701419	-0.3012576	-0.3374648	0.001084053	0.00019639	1.6211177	0.6969889	1.6211177	up	SERPINB7	8710	0010466 // negative	0005737 // cytoplasm //	0004867 // serine-type	chr18:61442608-61472612
11746201_a_at	0.39131546	0.27259064	0.31659222	-0.5003309	-0.38271952	-0.27259016	0.001554912	0.00066345	1.6381259	0.71204627	1.6381259	up	NAB1	4664	0001958 // endochondral	0005634 // nucleus // non-	0008134 // transcription	chr2:191513759-

11746408_a_at	0.39969015	0.26404858	0.2628255	-0.262825	-0.39101267	-0.3615799	0.001329397	0.00041087	1.5662638	0.64732724	1.5662638	up	SORT1	6272	0001503 // ossification //	0005634 // nucleus //	0004872 // receptor activity	chr1:109856530-
11731663_a_at	-0.27744627	-0.33054495	-0.3995843	0.320755	0.27744627	0.37389326	0.0009362	0.0001262	-1.579962	-0.65989	1.5799621	down	KIAA1217	56243	0007275 // multicellular	0005737 // cytoplasm //		chr10:24497595-24836772
11749633_a_at	0.25289965	0.23826933	0.3322296	-0.6304426	-0.2709465	-0.23826933	0.008314368	0.00711387	1.5739092	0.6543523	1.5739092	up	CLOCK	9575	0000077 // DNA damage	0005634 // nucleus //	0000978 // RNA	chr4:56300993-56412259 (-
11729512_a_at	0.6199298	0.41739225	0.5742593	-0.41739225	-0.6040921	-0.5161028	0.001094654	0.00021123	2.0701323	1.0497229	2.0701323	up	CSF3	1440	0006955 // immune	0005576 // extracellular	0005125 // cytokine activity	chr17:38171629-38174065
11744415_s_at	-0.25427914	-0.30656672	-0.3410654	0.25427866	0.44989347	0.40748215	0.00138566	0.00048299	-1.592384	-0.67118853	1.5923843	down	MFSD6	54842	0055085 // transmembrane	0016020 // membrane //		chr2:191273080-
11758095_s_at	-0.44552135	-0.5285702	-0.55374146	0.46253204	0.5299759	0.44552135	0.000566972	1.8665E-05	-1.984287	-0.98862076	1.984287	down	FPR1	2357	0000187 // activation of	0005768 // endosome //	0004871 // signal	chr19:52249085-52249466
11759114_at	-0.387424	-0.69571495	-0.70163774	0.41643095	0.39578676	0.38742447	0.001562753	0.00067035	-1.992813	-0.9948063	1.992813	down	RPL37	100506548 ///	0000184 // nuclear-	0005622 // intracellular //	0003723 // RNA binding //	chr5:40825363-40835314 (-
11739093_a_at	-0.30216312	-0.24048328	-0.31999016	0.31843662	0.36666393	0.24048328	0.001044088	0.00017107	-1.511597	-0.5960735	1.5115969	down	AZIN1	51582	0006521 // regulation of	0005829 // cytosol //	0003824 // catalytic activity	chr8:103838535-
11729329_at	0.36245537	0.3023424	0.27698803	-0.27698803	-0.33175278	-0.31081486	0.000623617	3.2065E-05	1.5373516	0.62044716	1.5373516	up	EPB41L4B	54566	0031032 // actomyosin	0005737 // cytoplasm //	0005200 // structural	chr9:112002006-
11743892_a_at	0.66860294	0.34650087	0.25877047	-0.30087805	-0.310709	-0.25877047	0.005860705	0.00471038	1.641192	0.714744	1.641192	up	ZNF780A	284323	0006351 // transcription,	0005622 // intracellular //	0003676 // nucleic acid	chr19:40538111-40596810
11762067_at	-0.32484198	-0.3033738	-0.4557538	0.40972614	0.30337334	0.6500468	0.002977789	0.00195664	-1.760158	-0.8157053	1.7601584	down	FCF1	51077	0006364 // rRNA	0005634 // nucleus //		chr14:75179884-75203497
11746229_a_at	-0.2601924	-0.37675428	-0.51041126	0.2601924	0.336843	0.35642767	0.00175734	0.00085288	-1.624813	-0.70027363	1.624813	down	RPL31	6160	0000184 // nuclear-	0005622 // intracellular //	0003723 // RNA binding //	chr2:101618750-
11720895_at	0.33594513	0.32602406	0.23742962	-0.31033802	-0.2374301	-0.32721567	0.000960295	0.00014359	1.5067717	0.5914608	1.5067717	up	SOS1	6654	0006915 // apoptotic	0005622 // intracellular //	0003677 // DNA binding //	chr2:39208540-39347604 (-
11727279_s_at	-0.37517452	-0.30295658	-0.43611717	0.4390583	0.3746109	0.30295658	0.001044088	0.00017333	-1.674377	-0.7436247	1.6743773	down	SLC4A7	9497	0006810 // transport //	0005886 // plasma	0005215 // transporter	chr3:27414213-27498245 (-
11723474_a_at	-0.26012564	-0.454422	-0.8543463	0.28071928	0.2601261	0.3056798	0.011280816	0.01010885	-1.747315	-0.8051397	1.747315	down	LYPLAL1	127018		0005737 // cytoplasm //	0004622 //	chr1:219347185-
11757459_s_at	-0.2509284	-0.3415885	-0.3400483	0.25092793	0.36814404	0.3808813	0.001105608	0.00022748	-1.562843	-0.6441728	1.562843	down	WIPF1	7456	0006461 // protein complex	0005737 // cytoplasm //	0003779 // actin binding //	chr2:175424301-
11717342_a_at	-0.47665787	-0.51082134	-0.44829798	0.4886484	0.44829845	0.49375343	0.000328584	2.04E-06	-1.939242	-0.9554925	1.9392415	down	SUPT4H1	6827	0000122 // negative	0005634 // nucleus //	0003700 // sequence-	chr17:56422538-56430454
11742759_a_at	0.4256773	0.46050167	0.25407124	-0.25464582	-0.26812553	-0.2540717	0.001446652	0.00056422	1.5572829	0.6390311	1.5572829	up	TRIP11	9321	0000042 // protein targeting	0005634 // nucleus //	0003713 // transcription	chr14:92435157-92506817
11739261_s_at	-0.265069	-0.26113796	-0.37444973	0.26113796	0.29375458	0.41475868	0.00138566	0.00047468	-1.54054	-0.623436	1.5405399	down	ACSL3	2181	0006629 // lipid metabolic	0005739 // mitochondrion //	0000166 // nucleotide	chr2:223725659-
11750076_a_at	-0.295269	-0.24726295	-0.42811775	0.36039782	0.24726295	0.2789855	0.001515835	0.00062846	-1.535915	-0.61909866	1.5359153	down	SNAP25	6616	0001504 // neurotransmitter	0005625 // soluble fraction //	0000149 // SNARE binding	chr20:10199520-10286856
11724967_at	0.4132042	0.46999788	0.28864193	-0.36162567	-0.30647802	-0.28864193	0.001129195	0.00025444	1.6352712	0.7095299	1.6352712	up	USP31	57478	0006508 // proteolysis //		0004221 // ubiquitin	chr16:23072728-23160591
11724014_a_at	-0.43079662	-0.32934475	-0.5284271	0.4391327	0.48221397	0.32934475	0.001229387	0.00032139	-1.798034	-0.84642	1.7980336	down	SOAT1	6646	0006629 // lipid metabolic	0005783 // endoplasmic	0000062 // fatty-acyl-CoA	chr1:179262934-
11718123_at	-0.20970821	-0.34887648	-0.4796586	0.3330474	0.20970869	0.26546478	0.003014235	0.00199183	-1.532076	-0.61548805	1.5320762	down	AIMP1	9255	0001525 // angiogenesis //	0005576 // extracellular	0000049 // tRNA binding //	chr4:107236852-
11728647_at	-0.31889153	-0.31509304	-0.4387045	0.3978529	0.31509304	0.34239292	0.000918864	0.00011546	-1.635059	-0.7093426	1.6350589	down	NUCKS1	64710	0016310 // phosphorylation	0005634 // nucleus //	0016301 // kinase activity //	chr1:205681946-
11730952_a_at	0.2745304	0.34665632	0.2891798	-0.32371092	-0.2745304	-0.2953248	0.000581859	2.1427E-05	1.5170944	0.60131085	1.5170944	up	PDK3	5165	0000160 // two-component	0005739 // mitochondrion //	0000155 // two-component	chrX:24483343-24558207
11720431_s_at	-0.38059473	-0.66845703	-0.7492614	0.38059473	0.46081257	0.48198366	0.00175734	0.00085637	-2.057037	-1.040568	2.0570374	down	COL15A1	1306	0001525 // angiogenesis //	0005576 // extracellular	0005198 // structural	chr9:101705920-
11760107_a_at	0.4245553	0.47408438	0.31304216	-0.33005142	-0.6318393	-0.31304216	0.002924236	0.00189919	1.7762955	0.8288716	1.7762955	up	PHF10	55274	0006351 // transcription,	0005634 // nucleus //	0008270 // zinc ion binding	chr6:170106835-
11721744_a_at	-0.2865219	-0.46196747	-0.49981022	0.3224783	0.35857296	0.2865219	0.001349742	0.00042951	-1.668584	-0.7386243	1.668584	down	NID2	22795	0007155 // cell adhesion //	0005576 // extracellular	0005509 // calcium ion	chr14:52471522-52535946
11722733_x_at	0.42790318	0.5006237	0.2039547	-0.2039547	-0.27702904	-0.25728512	0.003507549	0.002463	1.5406973	0.6235835	1.5406973	up	APPL1	26060	0006915 // apoptotic	0005634 // nucleus //	0005515 // protein binding //	chr3:57261764-57307486
11759667_a_at	-0.2701645	-0.2988634	-0.34884405	0.2701645	0.4342227	0.3134551	0.001170338	0.00028527	-1.563997	-0.64523804	1.5639973	down	TTC17	55761				chr11:43380480-43465760
11722368_a_at	-0.2300272	-0.28311634	-0.34582758	0.32794237	0.23002672	0.46104574	0.00204692	0.00111749	-1.543275	-0.6259953	1.5432751	down	TRIM22	10346	0006351 // transcription,	0005622 // intracellular //	0003700 // sequence-	chr11:5710839-5732093 (+)
11716332_a_at	-0.33304596	-0.4170127	-0.39243317	0.38403893	0.361763	0.33304596	0.000566128	1.391E-05	-1.670693	-0.74044657	1.6706929	down	RPL28	6158	0000184 // nuclear-	0005622 // intracellular //	0003723 // RNA binding //	chr19:55896712-55903460
11744456_s_at	0.38211918	0.3378892	0.2789235	-0.35721922	-0.464921	-0.2789235	0.001273598	0.000343	1.6245031	0.6999985	1.6245031	up	GSK3B	2932	0001837 // epithelial to	0005634 // nucleus //	0000166 // nucleotide	chr3:119545240-
11763206_at	-0.37801552	-0.3311534	-0.54567385	0.3311534	0.41976023	0.34996605	0.001296612	0.00036924	-1.72338	-0.7852408	1.72338	down	SH3RF2	153769	0010923 // negative		0004864 // protein	chr5:145317439-
11718766_at	-0.33440495	-0.27085495	-0.35589123	0.28129673	0.42092133	0.27085495	0.001186508	0.00029646	-1.563459	-0.64474136	1.5634589	down	PRSS23	11098	0006508 // proteolysis //	0005576 // extracellular	0003824 // catalytic activity	chr11:86511281-86522275
11721621_a_at	0.4630208	0.5346513	0.452878	-0.64771557	-0.56292725	-0.45287848	0.00080118	7.456E-05	2.053413	1.0380237	2.053413	up	KATNAL1	84056		0005737 // cytoplasm //	0000166 // nucleotide	chr13:30776629-30881653
11716596_a_at	-0.32248878	-0.38626003	-0.46557903	0.38395786	0.32248878	0.32994366	0.000860012	8.6482E-05	-1.666598	-0.73690605	1.6665978	down	SLC44A1	23446	0006810 // transport //	0005739 // mitochondrion //	0015220 // choline	chr9:108006905-
11726808_at	0.39615345	0.40161133	0.29172325	-0.37527657	-0.30486727	-0.29172325	0.000894235	9.9718E-05	1.6100644	0.68711835	1.6100644	up	ZAK	51776	0000075 // cell cycle	0005634 // nucleus //	0000166 // nucleotide	chr2:173940564-
11757500_a_at	-0.35916758	-0.4281788	-0.60201836	0.35916758	0.4524789	0.37808418	0.00130153	0.00037869	-1.814659	-0.8596984	1.8146589	down	CTDSPL	10217	0006470 // protein	0005625 // soluble fraction //	0004721 // phosphoprotein	chr3:38025592-38025959
11731513_at	-0.3393607	-0.27057648	-0.3447857	0.6510148	0.41264248	0.27057695	0.003600609	0.00255272	-1.696999	-0.7629857	1.696999	down	XPO4	64328	0006810 // transport //	0005634 // nucleus //	0005515 // protein binding //	chr13:21351468-21476914
11759499_at	-0.45605135	-0.48358727	-0.7768073	0.45605183	0.66595936	0.48368216	0.001714677	0.00081527	-2.15454	-1.1073797	2.1545396	down	FAM49A	81553		0005622 // intracellular //		chr2:16730588-16847599 (-
11715873_a_at	-0.39321232	-0.44879103	-0.49641562	0.4560957	0.40193558	0.39321232	0.000566972	1.7401E-05	-1.819095	-0.8632209	1.819095	down	OLFML3	56944	0007275 // multicellular	0005576 // extracellular		chr1:114521620-
11757828_s_at	-0.2821703	-0.33640862	-0.43728876	0.28216982	0.40849876	0.38795185	0.001174463	0.00028914	-1.637501	-0.711496	1.6375012	down	ATG5	9474	0000045 // autophagic	0005737 // cytoplasm //	0005515 // protein binding //	chr6:106632284-
11732132_a_at	-0.36360455	-0.36200523	-0.38748884	0.3692217	0.36200428	0.43048	0.000465863	5.2551E-06	-1.691459	-0.7582682	1.691459	down	IDS	3423	0008152 // metabolic	0005764 // lysosome //	0003824 // catalytic activity	chrX:148560300-
11741423_a_at	0.37368488	0.43097925	0.323668	-0.67333984	-0.6574302	-0.32366848	0.002398829	0.00142144	1.9020963	0.92759025	1.9020963	up	MIER1	57708	0006351 // transcription,	0005634 // nucleus //	0003677 // DNA binding //	chr1:67390577-67454302
11719916_at	0.7835617	0.426054	0.7157669	-0.6115322	-0.426054	-0.50594807	0.001583436	0.00069322	2.2288594	1.1563056	2.2288594	up	IL1B	3553	0000165 // MAPK cascade	0005576 // extracellular	0005125 // cytokine activity	chr2:113587327-
11725746_a_at	0.40911865	0.40372372	0.39000797	-0.42124462	-0.39000797	-0.47823524	0.000516757	6.1206E-06	1.778646	0.8307794	1.778646	up	PITPNC1	26207	0006810 // transport //	0005622 // intracellular //	0005515 // protein binding //	chr17:65373574-65690442
11739910_a_at	0.4014368	0.55310917	0.5276823	-0.4014368	-0.45498657	-0.5633602	0.000948463	0.00013253	1.9552286	0.9673373	1.9552286	up	ELOVL6	79071	0006633 // fatty acid	0005783 // endoplasmic	0005515 // protein binding //	chr4:110970228-
11731076_a_at	0.39556837	0.25215387	0.32160902	-0.25215435	-0.29451323	-0.46751547	0.001972237	0.00105164	1.5813662	0.66117144	1.5813662	up	LONRF1	91694	0006508 // proteolysis //		0004176 // ATP-dependent	chr8:12579414-12612999 (-
11729134_s_at	-0.30326366	-0.31749344	-0.31651592	0.3096714	0.32950878	0.30326366	0.000290487	2.7084E-07	-1.543893	-0.62657225	1.5438925	down	STK24	8428	0006468 // protein	0005634 // nucleus //	0000166 // nucleotide	chr13:99102454-99229400
11742378_a_at	-0.20807457	-0.38458014	-0.54796314	0.30126238	0.20807457	0.2598052	0.004418526	0.00333196	-1.554647	-0.63658667	1.5546466	down	AKR1B15	441282 /// 57016	0006081 // cellular aldehyde	0005576 // extracellular	0001758 // retinal	chr7:134252908-
11724488_at	-0.3146124	-0.59693575	-0.47804785	0.4344406	0.40684032	0.3146124	0.001579334	0.00069066	-1.800623	-0.84849644	1.8006233	down	AKR1B10	57016	0006081 // cellular aldehyde	0005576 // extracellular	0001758 // retinal	chr7:134212178-
11715353_s_at	-0.28896236	-0.4183712	-0.37634373	0.31534004	0.28896236	0.31404305	0.000787571	6.8902E-05	-1.588143	-0.6673409	1.5881431	down	COL1A2	1278	0001501 // skeletal system	0005576 // extracellular	0005201 // extracellular	chr7:94023872-94060539
11736928_a_at	0.40166235	0.40887213	0.27808523	-0.28195095	-0.27808523	-0.28499603	0.000894235	0.00011012	1.5632523	0.6445506	1.5632523	up	BMP2K	55589	0006468 // protein	0005634 // nucleus //	0000166 // nucleotide	chr4:79697531-79833339
11731518_at	-0.26633787	-0.2985487	-0.39362144	0.28234386	0.3911276	0.26633835	0.001226476	0.00031994	-1.550542	-0.6327726	1.550542	down	MFAP3	4238		0005886 // plasma		chr5:153418558-
11729157_a_at	-0.21583891	-0.49280834	-0.36879063	0.21583891	0.24988604	0.31659222	0.002934558	0.00191168	-1.536788	-0.61991835	1.5367882	down	MFAP4	4239	0007155 // cell adhesion //	0001527 // microfibril // non-	0005102 // receptor binding	chr17:19286754-19290503
11749356_a_at	0.360425	0.267529	0.2973261	-0.2675295	-0.41810083	-0.32607508	0.001105608	0.00023603	1.5644568	0.64566183	1.5644568	up	DCUN1D4	23142		0005634 // nucleus //		chr4:52709744-52779784
11717297_at	-0.28684092	-0.26835632	-0.45652103	0.42151356	0.29668093	0.26835632	0.001847473	0.00094145	-1.586766	-0.66608965	1.5867662	down	LRPPRC	10128	0006351 // transcription,	0000794 // condensed	0003677 // DNA binding //	chr2:44113362-44223144 (-
11729104_x_at	-0.355309	-0.30794716	-0.6176872	0.32477474	0.46122885	0.30794716	0.00285765	0.00183499	-1.731031	-0.79163134	1.7310307	down	UBA52	7311	0000075 // cell cycle	0005634 // nucleus //	0003735 // structural	chr19:18682613-18688360
11722864_at	-0.28141308	-0.26749754	-0.45053053	0.33571434	0.30177402	0.26749802	0.001411979	0.00051409	-1.552732	-0.63480914	1.5527323	down	NETO2	81831		0016020 // membrane //	0004872 // receptor activity	chr16:47115430-47177911
11722811_s_at	-0.32574654	-0.3784132	-0.37355518	0.4684677	0.32574654	0.43429947	0.000805831	7.6356E-05	-1.703785	-0.76874286	1.7037845	down	RAB7A	7879	0006184 // GTP catabolic	0005764 // lysosome //	0000166 // nucleotide	chr3:128444978-
11734458_a_at	0.46337795	0.53150463	0.4650178	-0.46337795	-0.9037852	-0.6993642	0.00170569	0.00080955	2.258674	1.175476	2.258674	up	FAM160B1	57700				chr10:116581502-
11754764_a_at	-0.25343132	-0.34517717	-0.38939524	0.4246421	0.25343132	0.38233423	0.00138566	0.00047061	-1.605256	-0.6828038	1.6052564	down	RAB7B	RAB7B	338382	0006810 // transport //	0005764 // lysosome //	0000166 // nucleotide
11743306_at	0.42589235	0.8004818	0.6442313	-0.5738106	-0.4258921	-0.52750254	0.00152408	0.00063899	2.1925406	1.1326036	2.1925406	up	FHDC1	85462	0016043 // cellular		0003779 // actin binding //	chr4:153864134-
11743677_at	0.55572605	0.36767006	0.46492195	-0.4901495	-0.36767006	-0.7756672	0.002596154	0.00160704	2.0101016	1.0072683	2.0101016	up	P2RY8	286530	0007165 // signal	0005886 // plasma	0004871 // signal	chrX:1581468-1656037 (-)
11725787_a_at	-0.5109968	-0.3956399	-0.4385891	0.39563942	0.43677044	0.45022678	0.000575727	1.9808E-05	-1.835221	-0.87595415	1.8352214	down	TMA16	55319				chr4:164415612-
11764037_s_at	-0.32436657	-0.31002045	-0.3483286	0.31341743	0.31002045	0.3354311	0.000309231	1.2089E-06	-1.56612	-0.64719486	1.5661201	down	DNAJA1	3301	0006457 // protein folding //	0016020 // membrane //	0005524 // ATP binding //	chr9:33039337-33039898
11742470_a_at	-0.30733156	-0.21765566	-0.5376272	0.27125216	0.35339785	0.21765566	0.004635097	0.00353318	-1.552909	-0.63497335	1.552909	down	SLC35F4	341880	0006810 // transport //	0016020 // membrane //		chr14:58030639-58063615
11716136_s_at	-0.34528923	-0.38497543	-0.4389887	0.41456604	0.34528923	0.54419136	0.001100865	0.00021337	-1.770839	-0.8244333	1.7708393	down	TMEM30A	55754	0036010 // protein	0005783 // endoplasmic	0005515 // protein binding //	chr6:75962639-75994706 (-
11729128_at	-0.54436207	-0.41387844	-0.5756769	0.48738384	0.6480465	0.41387844	0.001152694	0.00026912	-2.038831	-1.0277421	2.038831	down	CPA4	51200	0006508 // proteolysis //	0005576 // extracellular	0004180 //	chr7:129932973-
11721365_a_at	0.27109528	0.24074268	0.23177862	-0.23177862	-0.5576267	-0.2727146	0.005438874	0.00430079	1.5177269	0.6019122	1.5177269	up	IREB2	3658	0006417 // regulation of	0005737 // cytoplasm //	0003723 // RNA binding //	chr15:78730517-78793793
11715329_at	-0.25966644	-0.32529783	-0.44657755	0.36035204	0.32247305	0.25966644	0.001375919	0.00044966	-1.577906	-0.6580111	1.5779058	down	SLC6A15	55117	0006810 // transport //	0005886 // plasma	0005298 // proline:sodium	chr12:85274630-85306606
11730457_a_at	0.3608513	0.29745102	0.3119564	-0.4210453	-0.29745102	-0.40751266	0.000860012	8.8511E-05	1.6231045	0.69875586	1.6231045	up	AIM2	9447	0006955 // immune	0005634 // nucleus //	0003690 // double-stranded	chr1:159032040-
11733928_a_at	-0.3319044	-0.38096952	-0.49311686	0.44884825	0.3319044	0.40455627	0.001044088	0.00016955	-1.737605	-0.79709995	1.7376047	down	ARMC8	25852				chr3:137906147-
11760209_a_at	-0.29619598	-0.21833086	-0.25056887	0.54299545	0.21833086	0.4940381	0.003970606	0.00288334	-1.594923	-0.6734867	1.5949229	down	RPL37A	6168	0000184 // nuclear-	0005622 // intracellular //	0003735 // structural	chr2:217363564-
11737053_s_at	-0.496912	-0.22052002	-0.35803223	0.31842804	0.22051907	0.25288296	0.002858268	0.00183638	-1.539468	-0.6224314	1.5394675	down	HSPD1	3329	0002368 // B cell cytokine	0005615 // extracellular	0000166 // nucleotide	chr2:198351309-
11730928_a_at	-0.2255826	-0.41286516	-0.34011173	0.24433899	0.22558212	0.3453908	0.001723099	0.00082245	-1.513572	-0.59795713	1.5135719	down	IKBKB	3551	0002224 // toll-like receptor	0005634 // nucleus //	0000166 // nucleotide	chr8:42128819-42189956
11728991_a_at	-0.27641678	-0.25396585	-0.36181545	0.25396585	0.30201387	0.63941765	0.00637966	0.00519584	-1.619856	-0.69586515	1.6198555	down	IGDCC4	57722		0005886 // plasma		chr15:65673821-65715410
11755809_a_at	-0.4535246	-0.35923195	-0.61421776	0.4314561	0.35923195	0.3664894	0.001301856	0.00038029	-1.81678	-0.8613839	1.8167802	down	NBN	4683	0000077 // DNA damage	0000781 // chromosome,	0003684 // damaged DNA	chr8:90946680-90996858 (-
11719777_at	-0.32360888	-0.31502295	-0.60973835	0.41154957	0.31502247	0.42867613	0.002461901	0.00147811	-1.742557	-0.8012061	1.7425573	down	NDST1	3340	0000165 // MAPK cascade	0000139 // Golgi membrane	0003824 // catalytic activity	chr5:149887673-
11723591_at	0.46488428	0.4983406	0.4336486	-0.4336486	-0.47123766	-0.53656626	0.000556548	1.1647E-05	1.9266689	0.94610864	1.9266689	up	LRRC8C	84230		0005783 // endoplasmic		chr1:90098643-90185094
11721843_a_at	0.41340542	0.38862896	0.2963915	-0.32675648	-0.2963915	-0.37168503	0.000802412	7.5336E-05	1.6219765	0.69775295	1.6219765	up	ATL3	25923	0006184 // GTP catabolic	0005783 // endoplasmic	0000166 // nucleotide	chr11:63391553-63439385
11755211_a_at	0.47553253	0.6263027	0.43072414	-0.44372702	-0.5226264	-0.43072414	0.00092325	0.00011971	1.9677482	0.97654563	1.9677482	up	DICER1	23405	0000122 // negative	0005622 // intracellular //	0000166 // nucleotide	chr14:95556663-95603165
11758000_s_at	0.32646656	0.3142376	0.20053482	-0.20053482	-0.41100883	-0.32992554	0.002213554	0.00125472	1.509673	0.5942361	1.509673	up	CXADR	1525	0007005 // mitochondrion	0001669 // acrosomal	0004872 // receptor activity	chr21:18938820-18939264
11752508_a_at	-0.25358486	-0.35286236	-0.3376398	0.36537552	0.39239597	0.25358486	0.001108536	0.0002423	-1.571143	-0.65181446	1.5711429	down	HNRPLL	92906	0006397 // mRNA	0005634 // nucleus //	0000166 // nucleotide	chr2:38791173-38829999 (-
11754643_a_at	0.41511536	0.43558645	0.2952304	-0.34149408	-0.2952304	-0.36080217	0.000922398	0.00011768	1.6408988	0.7144863	1.6408988	up	TMEM234	56063		0016020 // membrane //		chr1:32681801-32683349 (-
11764247_s_at	-0.4097159	-0.8459561	-0.31874895	0.31874895	0.3732972	0.3873639	0.006893319	0.00572111	-1.846266	-0.8846103	1.8462658	down	WISP1	8840	0001558 // regulation of cell	0005576 // extracellular	0005520 // insulin-like	chr8:134242136-
11753446_x_at	-0.5495949	-0.22858906	-0.41397953	0.22858906	0.25733566	0.31347466	0.003363768	0.00232314	-1.58431	-0.6638543	1.5843096	down	HMOX1	3162	0001525 // angiogenesis //	0005615 // extracellular	0004392 // heme	chr22:35779095-35789589
11730623_at	-0.32889652	-0.32889652	-0.39707708	0.36363316	0.355608	0.32889652	0.000534553	9.6782E-06	-1.625634	-0.7010026	1.6256341	down	DSEL	92126		0016020 // membrane //	0008146 // sulfotransferase	chr18:65173820-65184217

11717924_a_at	0.36578178	0.35552883	0.27756023	-0.2775607	-0.3280511	-0.35510874	0.000719702	5.3953E-05	1.5726495	0.6531971	1.5726495	up	YAP1	10413	0001570 // vasculogenesis	0005634 // nucleus //	0001076 // RNA	chr11:101981209-
11731881_a_at	0.2632985	0.35311222	0.36092615	-0.35438585	-0.26329803	-0.29892826	0.000894235	0.00010409	1.5489776	0.6313163	1.5489776	up	PPP2R1B	5519	0006886 // intracellular	0030117 // membrane coat	0005515 // protein binding //	chr11:111597636-
11721643_at	-0.30627632	-0.36140442	-0.46140766	0.35037518	0.39424038	0.30627632	0.000981732	0.00015236	-1.654804	-0.72666013	1.6548038	down	ARL1	400	0006184 // GTP catabolic	0000139 // Golgi membrane	0000166 // nucleotide	chr12:101786911-
11726840_a_at	0.35307503	0.24781275	0.25596094	-0.24781275	-0.40020895	-0.29916334	0.001349742	0.00042926	1.5171299	0.6013446	1.5171299	up	AVL9	23080		0016020 // membrane //		chr7:32535059-32624403
11720682_at	-0.35052395	-0.28601217	-0.45834684	0.29760838	0.3595562	0.28601265	0.001118466	0.00025059	-1.601422	-0.6793534	1.6014218	down	TIFA	92610	0007249 // I-kappaB		0005515 // protein binding //	chr4:113196445-
11744150_s_at	-0.21227074	-0.36573553	-0.51121664	0.23833704	0.21227121	0.3232417	0.003618993	0.00257085	-1.537967	-0.62102425	1.5379667	down	LPIN2	9663	0006351 // transcription,	0005634 // nucleus //	0003713 // transcription	chr18:2916991-3011945 (-)
11720782_a_at	0.35474873	0.2146635	0.29564285	-0.29182673	-0.21466303	-0.44325972	0.002511213	0.0015233	1.52091	0.6049348	1.52091	up	CDK2	1017	0000075 // cell cycle	0000307 // cyclin-	0000166 // nucleotide	chr12:56360552-56366568
11716923_a_at	0.3008895	0.24064541	0.24355412	-0.4934826	-0.47126722	-0.24064589	0.002309014	0.00133853	1.583915	0.66349494	1.583915	up	PURB	5814	0006351 // transcription,	0005634 // nucleus //	0000900 // translation	chr7:44915850-44924985 (-
11716468_s_at	-0.30962086	-0.32732868	-0.5044298	0.36219788	0.38355732	0.30962086	0.001298686	0.00037457	-1.66123	-0.73225176	1.66123	down	ISCA1	81689	0016226 // iron-sulfur	0005739 // mitochondrion //	0005198 // structural	chr9:88879460-88897676 (-
11739642_a_at	0.5034838	0.44315434	0.44769764	-0.4431548	-0.4881091	-0.4837737	0.000344618	2.624E-06	1.9138235	0.93645775	1.9138235	up	STXBP5	134957	0006810 // transport //	0005737 // cytoplasm //	0005515 // protein binding //	chr6:147525507-
11717397_a_at	-0.2769866	-0.36006117	-0.46382856	0.37218046	0.28565454	0.2769866	0.001303801	0.00039599	-1.600548	-0.678566	1.600548	down	DDB2	1643	0000209 // protein	0005634 // nucleus //	0003677 // DNA binding //	chr11:47236492-47260768
11725305_a_at	0.32118416	0.4218092	0.25758934	-0.25758934	-0.4016266	-0.34486675	0.001381386	0.00046294	1.5891131	0.66822183	1.5891131	up	CPSF6	11052	0006378 // mRNA	0005634 // nucleus //	0000166 // nucleotide	chr12:69633316-69667892
11722316_at	-0.19079685	-0.32258034	-0.5963073	0.2818246	0.19079685	0.26292706	0.008603072	0.00740246	-1.53164	-0.6150777	1.5316404	down	SNX13	23161	0006810 // transport //	0005622 // intracellular //	0008289 // lipid binding //	chr7:17830384-17980141 (-
11716149_a_at	-0.22721195	-0.29267216	-0.38879347	0.35151052	0.22721195	0.3021369	0.001432485	0.00054657	-1.512057	-0.5965123	1.5120568	down	PMEPA1	56937	0030521 // androgen	0005886 // plasma	0050699 // WW domain	chr20:56223451-56285063
11741000_x_at	-0.2895012	-0.32375908	-0.43386173	0.2895012	0.33381557	0.42106724	0.001167238	0.00027881	-1.62132	-0.69716865	1.6213198	down	GAP43	2596	0007205 // protein kinase	0005886 // plasma	0005515 // protein binding //	chr3:115342150-
11716392_s_at	-0.38166428	-0.3907404	-0.4841051	0.4647646	0.43176746	0.38166428	0.000622499	3.1774E-05	-1.796143	-0.84490204	1.7961428	down	ADH5	128	0001523 // retinoid	0005634 // nucleus //	0000166 // nucleotide	chr4:99992379-100009939
11760540_at	-0.25883484	-0.36490583	-0.5981817	0.29187536	0.25883484	0.32420206	0.003418166	0.00237324	-1.623317	-0.6989449	1.6233172	down	PTCHD4	442213		0016020 // membrane //	0008158 // hedgehog	chr6:47868630-48036389 (-
11718862_at	-0.35477448	-0.26284218	-0.49582624	0.3988037	0.32460546	0.26284218	0.001765377	0.00086747	-1.62439	-0.6998981	1.6243901	down	SEC63	11231	0006457 // protein folding //	0005783 // endoplasmic	0004872 // receptor activity	chr6:108188961-
11725710_a_at	-0.5384517	-0.4018464	-0.7385411	0.54693127	0.56369495	0.4018464	0.001537282	0.00064989	-2.090388	-1.0637705	2.0903876	down	LOC100288602	100288602 ///	0000413 // protein peptidyl-	0005576 // extracellular	0003755 // peptidyl-prolyl	chr7:44836240-44842721
11720520_s_at	-0.39460564	-0.34552765	-0.48924828	0.34552765	0.40559578	0.41630173	0.00080118	7.3737E-05	-1.739817	-0.79893553	1.7398169	down	LOC728802	728802 /// 9659	0043623 // cellular protein	0005634 // nucleus //	0005515 // protein binding //	chr1:144851426-
11716153_a_at	0.41831875	0.39306736	0.24247074	-0.30358028	-0.2612543	-0.24247074	0.001349742	0.00042683	1.537288	0.6203874	1.537288	up	PAFAH1B1	5048	0000086 // G2/M transition	0000235 // astral	0005515 // protein binding //	chr17:2496923-2588911 (+)
11758770_at	-0.2795396	-0.3436017	-0.41283274	0.35287714	0.2795396	0.47892332	0.001411466	0.00050969	-1.642361	-0.7157713	1.642361	down	HSPA4	3308	0006950 // response to	0005634 // nucleus //	0000166 // nucleotide	chr5:132387658-
11722423_a_at	-0.26343966	-0.3350234	-0.41655302	0.33950758	0.26343918	0.3154831	0.001085089	0.00020247	-1.563178	-0.644482	1.563178	down	NEDD4L	23327	0006464 // cellular protein	0005622 // intracellular //	0004842 // ubiquitin-protein	chr18:55711607-56068771
11725931_at	-0.24481058	-0.46965122	-0.48487568	0.31368065	0.32027388	0.24481058	0.001962186	0.00103975	-1.616307	-0.69270086	1.6163065	down	HSPA5	3309	0002576 // platelet	0005634 // nucleus //	0000166 // nucleotide	chr9:127997128-
11748751_a_at	-0.32633352	-0.45857334	-0.30366278	0.32118845	0.36648512	0.30366278	0.00104443	0.00018026	-1.61698	-0.693302	1.6169802	down	GPNMB	10457	0007155 // cell adhesion //	0005887 // integral to	0005178 // integrin binding //	chr7:23286386-23314370
11733088_at	-0.2890439	-0.42390394	-0.25018215	0.30491447	0.28462887	0.25018215	0.00131041	0.00039874	-1.516717	-0.60095185	1.516717	down	MAF	4094	0000122 // negative	0000785 // chromatin //	0003677 // DNA binding //	chr16:79627687-79634622
11752869_s_at	0.25792122	0.342144	0.36331367	-0.27279472	-0.26302862	-0.2579217	0.000719702	5.5611E-05	1.5007753	0.585708	1.5007753	up	CEBPD	1052	0006351 // transcription,	0005634 // nucleus //	0003677 // DNA binding //	chr8:48649475-48650977 (-
11758093_s_at	-0.5704503	-0.20408297	-0.60862446	0.20408249	0.28447247	0.27805138	0.006688939	0.00551052	-1.643291	-0.716588	1.643291	down	FBN1	2200	0001501 // skeletal system	0001527 // microfibril //	0005201 // extracellular	chr15:48700504-48700908
11741484_a_at	0.18179846	0.22910833	0.25468826	-0.39525604	-0.5567994	-0.18179846	0.006797905	0.00561891	1.5155236	0.5998163	1.5155236	up	SCRN1	9805	0006508 // proteolysis //	0005634 // nucleus //	0016805 // dipeptidase	chr7:29959719-30029425 (-
11726782_at	-0.44628143	-0.28987122	-0.457129	0.33632517	0.28987074	0.34666872	0.001103597	0.0002204	-1.649523	-0.72204876	1.6495229	down	C15orf29	79768		0005730 // nucleolus //		chr15:34432874-34502297
11732171_x_at	-0.605227	-0.43159676	-0.89450693	0.43159676	0.577477	0.4366598	0.002396185	0.00141614	-2.182056	-1.1256881	2.182056	down	GRSF1	2926	0006378 // mRNA	0005737 // cytoplasm //	0000166 // nucleotide	chr4:71682122-71705607 (-
11722518_a_at	0.4530573	0.453012	0.23250818	-0.2522149	-0.26588106	-0.23250818	0.001973392	0.00105377	1.5472723	0.6297272	1.5472723	up	SMC5	23137	0000722 // telomere	0000781 // chromosome,	0000166 // nucleotide	chr9:72873877-72969789
11721526_a_at	-0.56881523	-0.4924078	-0.73890257	0.5719285	0.5122237	0.4924078	0.000936118	0.00012527	-2.181865	-1.125562	2.1818652	down	PROSER1	80209				chr13:39584001-39612252
11739134_a_at	0.47004795	0.41727257	0.4473219	-0.55922747	-0.62589836	-0.4172721	0.000894235	0.00010256	1.971117	0.97901344	1.971117	up	COL5A2	1290	0001501 // skeletal system	0005576 // extracellular	0005102 // receptor binding	chr2:189896617-
11717272_at	-0.24949074	-0.36197758	-0.37938452	0.24949074	0.3822794	0.27579117	0.001301856	0.0003872	-1.550576	-0.6328047	1.5505764	down	COL5A1	1289	0001568 // blood vessel	0005576 // extracellular	0005178 // integrin binding //	chr9:137533565-
11716663_a_at	-0.3415699	-0.6031084	-0.3280506	0.3280506	0.39406204	0.4964304	0.00219438	0.00124047	-1.778208	-0.83042395	1.7782078	down	GDF15	9518	0007165 // signal	0005576 // extracellular	0005125 // cytokine activity	chr19:18496769-18499986
11727094_at	-0.23634768	-0.24107456	-0.4794693	0.3469162	0.24874401	0.2363472	0.003472815	0.00242886	-1.511834	-0.59629965	1.5118339	down	TMEM19	55266		0016020 // membrane //		chr12:72079866-72097836
11733501_a_at	-0.33056688	-0.42229033	-0.585165	0.3979597	0.3305664	0.34921265	0.00138566	0.00047509	-1.747453	-0.8052536	1.747453	down	ADCY8	114	0006112 // energy reserve	0005622 // intracellular //	0000166 // nucleotide	chr8:131792546-
11758066_s_at	-0.23281622	-0.32646513	-0.2788806	0.41062117	0.23281622	0.28004074	0.001490506	0.0005973	-1.502342	-0.5872134	1.5023421	down	VAPB	9217	0006665 // sphingolipid	0005783 // endoplasmic	0005198 // structural	chr20:57021492-57021961
11735374_a_at	-0.28822756	-0.5367174	-0.3541479	0.32356834	0.30109024	0.28822708	0.001644178	0.00074586	-1.621497	-0.6973262	1.6214968	down	GDF5	8200	0007179 // transforming	0005576 // extracellular	0005125 // cytokine activity	chr20:34021148-34026027
11762508_x_at	-0.39912796	-0.22487211	-0.3036499	0.22487211	0.27043152	0.34687996	0.001562196	0.0006678	-1.505189	-0.5899445	1.5051888	down	SLC35A3	23443	0006047 // UDP-N-	0000139 // Golgi membrane	0005338 // nucleotide-sugar	chr1:100435561-
11715778_a_at	-0.26840973	-0.32301235	-0.45174217	0.26840973	0.34627342	0.30275917	0.001298686	0.00037359	-1.573018	-0.65353554	1.5730184	down	LUZP6	136319 ///	0006417 // regulation of	0005634 // nucleus //		chr7:135611507-
11743863_a_at	-0.27326488	-0.30248642	-0.34323215	0.2732644	0.32648182	0.3548293	0.000660041	3.7498E-05	-1.541698	-0.6245197	1.5416975	down	ATP2B1	490	0006754 // ATP biosynthetic	0005886 // plasma	0000166 // nucleotide	chr12:89981825-90049887
11724435_a_at	0.6252079	0.4891534	0.3516636	-0.3516636	-0.36258936	-0.4515009	0.001407042	0.00049865	1.8368828	0.8772596	1.8368828	up	TPK1	27010	0006766 // vitamin	0005737 // cytoplasm //	0000166 // nucleotide	chr7:144149033-
11735329_a_at	-0.2893381	-0.26240826	-0.4345188	0.26764965	0.26240826	0.34186554	0.00138566	0.00047436	-1.536232	-0.6193962	1.5362321	down	CD47	961	0007155 // cell adhesion //	0005886 // plasma	0005515 // protein binding //	chr3:107761938-
11719186_a_at	-0.81661224	-0.48213768	-0.28837013	0.28837013	0.35748196	0.33519697	0.006508971	0.00532502	-1.810084	-0.8560564	1.8100836	down	N4BP2L2	10443				chr13:33090827-33112941
11721725_at	-0.2613163	-0.28415442	-0.30206537	0.2613163	0.41247368	0.37546778	0.001044088	0.00017656	-1.549996	-0.6322646	1.5499961	down	PSKH1	5681	0006468 // protein	0005634 // nucleus //	0000166 // nucleotide	chr16:67927164-67963581
11722573_a_at	-0.501564	-0.29663658	-0.32499695	0.44321156	0.29663658	0.350729	0.001557737	0.00066509	-1.667775	-0.73792493	1.6677753	down	ERAP2	64167	0002474 // antigen	0005783 // endoplasmic	0004177 // aminopeptidase	chr5:96211642-96255430
11720377_s_at	-0.35531378	-0.2664113	-0.46994543	0.35142946	0.31170988	0.2664113	0.001378478	0.00045565	-1.595203	-0.6737404	1.5952034	down	SAP30L	79685	0006351 // transcription,	0005634 // nucleus //	0003677 // DNA binding //	chr5:153825516-
11718384_at	-0.22359753	-0.30348396	-0.33772087	0.22359753	0.3532591	0.44011974	0.001833103	0.00092689	-1.544628	-0.6272596	1.5446281	down	C2orf68	388969				chr2:85832377-85839181 (-
11720614_s_at	-0.23377228	-0.2226696	-0.27938175	0.37006664	0.2226696	0.43115997	0.001695133	0.00079957	-1.501676	-0.58657324	1.5016756	down	CLNS1A	1207	0000387 // spliceosomal	0005634 // nucleus //	0005515 // protein binding //	chr11:77327196-77348845
11748854_a_at	0.452466	0.4785571	0.3052144	-0.40490675	-0.30521488	-0.48213053	0.001320748	0.00040487	1.7525998	0.8094966	1.7525998	up	RPUSD4	84881	0001522 // pseudouridine	0005739 // mitochondrion //	0003723 // RNA binding //	chr11:126072693-
11721419_at	-0.12838459	-0.5106001	-0.8887634	0.12838459	0.13949728	0.18901253	0.040671483	0.03982273	-1.581778	-0.66154754	1.5817784	down	POLI	11201	0006260 // DNA replication	0005622 // intracellular //	0003677 // DNA binding //	chr18:51795773-51824602
11724647_at	-0.38502932	-0.29494762	-0.6243286	0.29494762	0.46586227	0.40938854	0.002722581	0.00171524	-1.771332	-0.8248347	1.771332	down	VANGL1	81839	0007275 // multicellular	0016020 // membrane //	0005515 // protein binding //	chr1:116184554-
11720728_at	0.34445333	0.31653452	0.27149963	-0.5053034	-0.27149963	-0.38101244	0.001491167	0.00059798	1.6208692	0.6967677	1.6208692	up	UBR1	197131	0006511 // ubiquitin-	0000151 // ubiquitin ligase	0004842 // ubiquitin-protein	chr15:43235094-43398311
11756109_s_at	-0.45202732	-0.23437023	-0.31308174	0.27066517	0.302763	0.23437023	0.001729953	0.00082887	-1.518267	-0.60242593	1.5182674	down	MGAT2	4247	0006486 // protein	0000139 // Golgi membrane	0008455 // alpha-1,6-	chr14:50088317-50090037
11723420_s_at	0.57678556	0.519259	0.4977088	-0.7283616	-0.69699144	-0.4977088	0.000894235	0.00010301	2.2536628	1.1722717	2.2536628	up	BTF3L4	91408				chr1:52521801-52554585
11737812_a_at	-0.3495636	-0.23176384	-0.35951757	0.3282361	0.23176384	0.4117303	0.001538867	0.00065139	-1.555658	-0.6375251	1.5556582	down	NNT	23530	0006099 // tricarboxylic acid	0005739 // mitochondrion //	0000166 // nucleotide	chr5:43603228-43705667
11719283_a_at	0.40232277	0.29685307	0.26308537	-0.26308537	-0.34620094	-0.5117817	0.002125659	0.00118256	1.6182595	0.69444305	1.6182595	up	UBR7	55148	0016567 // protein		0004842 // ubiquitin-protein	chr14:93673400-93695560
11747742_a_at	0.29079962	0.3452978	0.39448118	-0.29079962	-0.36407566	-0.38058138	0.0007963	7.1636E-05	1.6118064	0.68867844	1.6118064	up	MTF2	22823		0005634 // nucleus //	0003677 // DNA binding //	chr1:93544797-93602349
11728288_a_at	0.362216	0.3335371	0.40118456	-0.49626207	-0.3335371	-0.37978077	0.000935536	0.00012395	1.7038983	0.76883924	1.7038983	up	KRT15	3866	0008544 // epidermis	0005882 // intermediate	0005198 // structural	chr17:39669998-39678176
11718572_s_at	-0.30665493	-0.27721214	-0.2671032	0.36820507	0.35116768	0.2671032	0.000719702	5.2188E-05	-1.528887	-0.6124821	1.5288873	down	SERBP1	26135	0043488 // regulation of	0005634 // nucleus //	0003723 // RNA binding //	chr1:67873494-67896452 (-
11742714_a_at	-0.32288313	-0.3056593	-0.64437056	0.33654833	0.3056593	0.39250517	0.003480045	0.00243682	-1.704335	-0.7692086	1.7043346	down	TBRG1	84897	0006260 // DNA replication	0005634 // nucleus //	0005515 // protein binding //	chr11:124492740-
11763357_a_at	-0.36782932	-0.44537163	-0.555078	0.45358944	0.36782932	0.42658043	0.000944286	0.00012969	-1.830316	-0.8720927	1.830316	down	TMEM170A	124491		0016020 // membrane //		chr16:75477141-75498587
11720520_s_at	-0.39460564	-0.34552765	-0.48924828	0.34552765	0.40559578	0.41630173	0.00080118	7.3737E-05	-1.739817	-0.79893553	1.7398169	down	PDE4DIP	728802 /// 9659	0043623 // cellular protein	0005634 // nucleus //	0005515 // protein binding //	chr1:144851426-
11716051_at	-0.37384987	-0.33125782	-0.5256796	0.44242287	0.33125782	0.51732826	0.001381386	0.00046429	-1.790793	-0.84059876	1.7907932	down	LAMC1	3915	0006461 // protein complex	0005576 // extracellular	0005201 // extracellular	chr1:182992594-
11717451_at	-0.5119424	-0.45357466	-0.55706406	0.45357466	0.5508604	0.46799135	0.000575727	1.9685E-05	-1.997694	-0.99833584	1.9976943	down	NEK7	140609	0000910 // cytokinesis //	0000922 // spindle pole //	0000166 // nucleotide	chr1:198126109-
11758797_s_at	-0.32431698	-0.32566118	-0.5270753	0.32431698	0.3611002	0.37655973	0.001336215	0.00041728	-1.677536	-0.7463435	1.6775358	down	SRSF10	10772	0000244 // assembly of	0005634 // nucleus //	0000166 // nucleotide	chr1:24290836-24306885 (-
11756171_a_at	-0.9235263	-0.7232671	-0.90455294	0.80094194	0.7232671	0.75068855	0.000566972	1.866E-05	-3.049871	-1.608748	3.0498705	down	PFKFB2	5208	0005975 // carbohydrate	0005737 // cytoplasm //	0000166 // nucleotide	chr1:207226384-
11721732_at	0.32659912	0.26837254	0.28953266	-0.28150988	-0.34985876	-0.26837254	0.00066257	4.0546E-05	1.5102093	0.5947485	1.5102093	up	TMX3	54495	0006662 // glycerol ether	0005783 // endoplasmic	0003756 // protein disulfide	chr18:66340924-66382535
11736144_at	-0.4046564	-0.33716345	-0.35963774	0.35557985	0.39895296	0.33716297	0.000555711	1.1069E-05	-1.659848	-0.73105115	1.659848	down	ENPP4	22875	0008152 // metabolic	0016020 // membrane //	0003824 // catalytic activity	chr6:46097691-46114436
11722922_s_at	-0.2761159	-0.2643571	-0.43359327	0.3133378	0.2643571	0.29350424	0.001314637	0.00040088	-1.531652	-0.61508846	1.5316519	down	FAM122B	159090				chrX:133903595-
11721766_at	-0.31434774	-0.3755164	-0.5364809	0.32804966	0.3645482	0.31434727	0.001302616	0.00039364	-1.675312	-0.74443007	1.6753123	down	ADRBK2	157	0006468 // protein		0000166 // nucleotide	chr22:25960815-26125261
11718788_a_at	-0.4442067	-0.48919106	-0.44741678	0.5593281	0.4442067	0.45734978	0.000566972	1.733E-05	-1.928171	-0.947233	1.928171	down	ADA	100	0001666 // response to	0005615 // extracellular	0001883 // purine	chr20:43248162-43280383
11723167_at	-0.371552	-0.32657862	-0.32299137	0.5784044	0.51615953	0.32299185	0.001396973	0.00048999	-1.75673	-0.81289256	1.7567301	down	EPHB2	2048	0001525 // angiogenesis //	0005886 // plasma	0000166 // nucleotide	chr1:23037256-23241822
11723200_s_at	-0.29266548	-0.22669601	-0.4193039	0.292943	0.22669601	0.31177616	0.00157929	0.00068591	-1.505275	-0.59002686	1.5052748	down	HNRNPA3	220988	0000398 // nuclear mRNA	0005634 // nucleus //	0000166 // nucleotide	chr2:178077022-
11732200_at	-0.3649249	-0.37892914	-0.39272213	0.59436464	0.36492538	0.5335121	0.001103597	0.00022241	-1.835864	-0.8764594	1.8358643	down	PLAGL2	5326	0006351 // transcription,	0005622 // intracellular //	0001077 // RNA	chr20:30780310-30795546
11725803_a_at	0.3269415	0.28448057	0.21487379	-0.21487379	-0.26116848	-0.8783183	0.029311648	0.02830318	1.6550623	0.7268855	1.6550623	up	C21orf91	54149				chr21:19161290-19191703
11718769_a_at	-0.48324823	-0.6816025	-0.7492566	0.48324776	0.5032673	0.65921736	0.001141132	0.00025938	-2.276178	-1.1866133	2.276178	down	LAMTOR3	8649	0016310 // phosphorylation	0005768 // endosome //	0005515 // protein binding //	chr4:100799494-
11729802_at	0.38630867	0.39304256	0.2909565	-0.3257966	-0.2909565	-0.2912364	0.000702939	4.6387E-05	1.5794611	0.6594324	1.5794611	up	EIF1AD	84285	0006413 // translational	0005634 // nucleus //	0003723 // RNA binding //	chr11:65764015-65769647
11756316_a_at	-0.40073395	-0.5390501	-0.41957092	0.5815716	0.40073395	0.58730984	0.001085089	0.00020119	-1.967445	-0.9763234	1.9674451	down	CHI3L1	1116	0005975 // carbohydrate	0005576 // extracellular	0003824 // catalytic activity	chr1:203148092-
11715509_a_at	0.35466003	0.3415966	0.3883772	-0.38046455	-0.4109211	-0.3415966	0.000534553	7.1025E-06	1.6692562	0.73920536	1.6692562	up	NDFIP1	80762	0002761 // regulation of	0000139 // Golgi membrane	0004871 // signal	chr5:141488069-
11733798_at	-0.7046766	-0.41445065	-0.95101833	0.6510148	0.41445112	0.53211355	0.002977789	0.00195627	-2.333629	-1.222575	2.3336287	down	DDX5	1655	0000122 // negative	0005634 // nucleus //	0000166 // nucleotide	chr17:62494373-62502477
11716291_s_at	-0.30240917	-0.3459177	-0.32229805	0.33730698	0.34051895	0.30240917	0.000398697	3.1482E-06	-1.56948	-0.6502867	1.5694801	down	HNRNPA0	10949	0000398 // nuclear mRNA	0005634 // nucleus //	0000166 // nucleotide	chr5:137086801-
11739834_s_at	-0.39600372	-0.22538757	-0.34540558	0.33333778	0.25536537	0.22538757	0.00147514	0.00058407	-1.509038	-0.59362924	1.5090381	down	HSPE1-MOB4	100529241 ///	0006810 // transport //	0005624 // membrane	0005515 // protein binding //	chr2:198380770-
11755498_s_at	-0.32226467	-0.44509888	-0.5551691	0.32226467	0.37949467	0.38100147	0.00124487	0.00033141	-1.743232	-0.8017645	1.7432319	down	ATP6V0E2	155066	0006810 // transport //	0005773 // vacuole //	0015078 // hydrogen ion	chr7:149571064-
11719149_a_at	-0.29242134	-0.26245117	-0.51474476	0.32692146	0.26820564	0.26245117	0.002427876	0.00144673	-1.560922	-0.64239854	1.5609221	down	SAP130	79595	0006351 // transcription,	0005634 // nucleus //	0003713 // transcription	chr2:128698795-
11719741_a_at	0.24753237	0.37866735	0.30831242	-0.26399326	-0.45351505	-0.24753237	0.002081092	0.00114442	1.5509845	0.63318425	1.5509845	up	DCP2	167227	0000184 // nuclear-	0000932 // cytoplasmic	0003723 // RNA binding //	chr5:112312398-
11733995_x_at	-0.22817469	-0.24793959	-0.29814482	0.38851595	0.22817469	0.43128777	0.001641907	0.00074039	-1.523524	-0.60741246	1.5235243	down	NADKD1	133686	0008152 // metabolic	0005739 // mitochondrion //	0003951 // NAD+ kinase	chr5:36192694-36242144 (-

11723638_at	-0.2705555	-0.42683744	-0.4953661	0.33410454	0.37555504	0.2705555	0.001476138	0.00058533	-1.652127	-0.7243247	1.6521271	down	C11orf58	10944				chr11:16759947-16779901
11721805_at	0.38227034	0.27483606	0.3016119	-0.27483606	-0.28495312	-0.2833681	0.00071607	5.008E-05	1.5163735	0.6006252	1.5163735	up	KLF2	10365	0000902 // cell	0005622 // intracellular //	0003676 // nucleic acid	chr19:16435649-16439053
11724411_a_at	-0.29523182	-0.34613037	-0.21161604	0.29516745	0.21161604	0.3998766	0.001850825	0.0009442	-1.501647	-0.58654606	1.5016474	down	PPARA	5465	0000122 // negative	0005634 // nucleus //	0001103 // RNA	chr22:46546730-46641621
11732455_at	-0.4330988	-0.4279542	-0.34581566	0.34581566	0.40139866	0.54800606	0.001105608	0.00023402	-1.782658	-0.8340297	1.7826576	down	KCNJ15	3772	0006810 // transport //	0005886 // plasma	0005216 // ion channel	chr21:39628796-39674361
11717903_s_at	0.28837872	0.35656738	0.38833904	-0.28837872	-0.3574133	-0.40879154	0.000894235	0.00010821	1.6199578	0.69595623	1.6199578	up	IER3IP1	51124	0042981 // regulation of	0005783 // endoplasmic		chr18:44681412-44702735
11733304_x_at	-0.37318707	-0.33692265	-0.41695404	0.48379803	0.33692265	0.39536476	0.000860012	8.7381E-05	-1.718381	-0.7810497	1.7183807	down	ZMAT3	64393	0006810 // transport //	0005622 // intracellular //	0003676 // nucleic acid	chr3:178741516-
11716245_at	-0.49366713	-0.4610319	-0.64034843	0.4897933	0.4610319	0.73947763	0.001381386	0.00046345	-2.136304	-1.0951169	2.136304	down	WASF2	10163	0001525 // angiogenesis //	0001726 // ruffle // inferred	0003779 // actin binding //	chr1:27731800-27816678 (-
11726184_at	-0.25174284	-0.30967665	-0.2981062	0.25174332	0.37731838	0.3157587	0.000922398	0.0001179	-1.517239	-0.60144866	1.5172393	down	ARSI	340075	0008152 // metabolic	0005576 // extracellular	0003824 // catalytic activity	chr5:149675901-
11719222_at	-0.7107172	-0.2842822	-0.6807661	0.2842822	0.37320662	0.45521784	0.004199299	0.00312283	-1.904604	-0.9294907	1.9046035	down	SPINK1	6690	0010466 // negative	0005576 // extracellular	0004866 // endopeptidase	chr5:147204145-
11723374_at	-0.23574257	-0.3592968	-0.37662458	0.23574257	0.25116014	0.3499875	0.001375919	0.00045205	-1.518715	-0.6028514	1.5187153	down	GLI3	2737	0000122 // negative	0005622 // intracellular //	0003676 // nucleic acid	chr7:42000549-42276796 (-
11734736_a_at	0.3018446	0.38690853	0.34512997	-0.32839155	-0.3018446	-0.36473036	0.000585138	2.4752E-05	1.5980176	0.67628324	1.5980176	up	FAR1	84188	0006629 // lipid metabolic	0005777 // peroxisome //	0000166 // nucleotide	chr11:13689891-13753889
11721284_a_at	0.48772573	0.33825684	0.32447624	-0.39832592	-0.32447624	-0.39496756	0.001075945	0.00019385	1.688891	0.7560762	1.688891	up	CDS2	8760	0008654 // phospholipid	0005739 // mitochondrion //	0004605 // phosphatidate	chr20:5107406-5172338 (+)
11716024_a_at	-0.52563	-0.29955292	-0.5614171	0.3963604	0.4438753	0.29955292	0.001695564	0.0008014	-1.792694	-0.8421296	1.7926944	down	GOLGA7	51125		0000139 // Golgi membrane		chr8:41348134-41368499
11734049_a_at	-0.3732462	-0.28305244	-0.47196913	0.34772873	0.28456593	0.28305244	0.001211694	0.00031232	-1.603478	-0.6812049	1.6034784	down	SEH1L	81929	0000087 // M phase of	0000775 // chromosome,		chr18:12947982-12987533
11722831_a_at	-0.65859175	-0.60082006	-0.8512478	0.6008196	0.74006176	0.6377821	0.000881439	9.4327E-05	-2.572387	-1.3631077	2.572387	down	CCNY	219771	0000079 // regulation of	0000308 // cytoplasmic	0005515 // protein binding //	chr10:35625801-35860854
11735045_at	-0.5342245	-0.39545918	-0.57310057	0.4184289	0.6139817	0.39545965	0.001296612	0.00037174	-1.968211	-0.97688484	1.9682109	down	ATP6V0D2	245972	0006810 // transport //	0005768 // endosome //	0005515 // protein binding //	chr8:87111065-87166457
11723470_a_at	0.39689398	0.32937098	0.24790382	-0.26230574	-0.24790335	-0.29050064	0.001071701	0.00019083	1.5069444	0.59162617	1.5069444	up	LPGAT1	9926	0008152 // metabolic	0005737 // cytoplasm //	0016740 // transferase	chr1:211916799-
11737223_a_at	0.39891052	0.30948496	0.3020587	-0.38914776	-0.3104086	-0.3020587	0.000860012	8.7323E-05	1.5918338	0.67068976	1.5918338	up	ARTN	9048	0007165 // signal	0005576 // extracellular	0005102 // receptor binding	chr1:44398991-44402912
11719096_at	-0.33930063	-0.44518757	-0.57995844	0.49441242	0.3393011	0.34133482	0.001498547	0.00061198	-1.798131	-0.8464983	1.7981312	down	CYB5B	80777	0006810 // transport //	0005739 // mitochondrion //	0008047 // enzyme	chr16:69458427-69500167
11740062_a_at	-0.28473854	-0.37228775	-0.36680603	0.40562725	0.32081604	0.28473854	0.00092325	0.00011961	-1.600295	-0.67833805	1.6002952	down	SIGMAR1	10280	0006696 // ergosterol	0005634 // nucleus //	0000247 // C-8 sterol	chr9:34634719-34637806 (-
11716233_a_at	0.28965425	0.31061506	0.29881334	-0.40198755	-0.28965425	-0.6244974	0.00270863	0.00169881	1.6683329	0.73840725	1.6683329	up	CTNNBIP1	56998	0000122 // negative	0005634 // nucleus //	0005515 // protein binding //	chr1:9908335-9970385 (-)
11736247_x_at	-0.23855734	-0.40548468	-0.43902683	0.23855782	0.25946712	0.34346485	0.001676314	0.00077807	-1.559971	-0.64151955	1.5599713	down	AMIGO2	347902	0006916 // anti-apoptosis //	0005634 // nucleus //	0005515 // protein binding //	chr12:47469489-47473734
11719680_x_at	-0.2964716	-0.35799313	-0.30198193	0.2964716	0.3421316	0.39783478	0.000705057	4.6892E-05	-1.584794	-0.6642949	1.5847936	down	TNFRSF10B	8795	0006915 // apoptotic	0005886 // plasma	0004872 // receptor activity	chr8:22877645-22926761 (-
11718295_a_at	0.2639923	0.39488316	0.2963462	-0.2639923	-0.36003494	-0.35339022	0.001094578	0.00021061	1.5628865	0.644213	1.5628865	up	SEMA4B	10509	0007275 // multicellular	0016020 // membrane //	0004872 // receptor activity	chr15:90728146-90772892
11725885_a_at	0.35227108	0.25337887	0.2768073	-0.3553357	-0.25337887	-0.3311777	0.000960295	0.0001438	1.5235637	0.6074498	1.5235637	up	CNEP1R1	255919	0006629 // lipid metabolic	0005634 // nucleus //	0005515 // protein binding //	chr16:50059135-50070992
11719366_s_at	0.5175524	0.45788383	0.69309425	-0.7759476	-0.45788383	-0.60954237	0.001428078	0.00053966	2.2511072	1.1706347	2.2511072	up	CXCL1	2919	0006935 // chemotaxis //	0005576 // extracellular	0005102 // receptor binding	chr4:74735118-74737019
11729600_a_at	-0.28327894	-0.23501635	-0.49890614	0.23501635	0.32481575	0.2393055	0.003196239	0.00216778	-1.521449	-0.60544634	1.5214493	down	PPP6C	5537	0000082 // G1/S transition	0005737 // cytoplasm //	0004721 // phosphoprotein	chr9:127908852-
11728477_at	0.9831128	0.60692406	0.72326326	-0.6630931	-0.60692406	-0.6941314	0.001105608	0.00023582	2.6866648	1.4258163	2.6866648	up	CXCL3	2921	0006935 // chemotaxis //	0005576 // extracellular	0005125 // cytokine activity	chr4:74902305-74904523 (-
11755593_a_at	-0.3178811	-0.31848192	-0.40737152	0.31788063	0.36052847	0.44405365	0.000894235	0.00010926	-1.649542	-0.72206575	1.6495422	down	ATG13	9776	0000045 // autophagic	0000407 // pre-	0005515 // protein binding //	chr11:46690953-46696367
11744127_at	0.7169781	0.3812685	0.68879986	-0.4513216	-0.41943932	-0.38126898	0.001656829	0.00075646	2.018139	1.0130254	2.018139	up	CXCL2	2920	0002237 // response to	0005576 // extracellular	0005125 // cytokine activity	chr4:74962676-74965002 (-
11722261_at	0.3709383	0.28147984	0.2415266	-0.31953335	-0.2415266	-0.3143854	0.001093891	0.00020977	1.5050347	0.5897967	1.5050347	up	EDEM1	9695	0006457 // protein folding //	0005783 // endoplasmic	0004571 // mannosyl-	chr3:5229250-5261650 (+)
11721733_a_at	-0.42862988	-0.19655514	-0.43253946	0.28977442	0.19655514	0.24694252	0.002959071	0.0019385	-1.512567	-0.5969989	1.5125668	down	GCH1	2643	0006184 // GTP catabolic	0005625 // soluble fraction //	0000166 // nucleotide	chr14:55308678-55370518
11719644_a_at	-0.23360968	-0.35651064	-0.53896236	0.3035488	0.2336092	0.36952496	0.003227019	0.00219685	-1.600573	-0.6785885	1.6005731	down	GATAD2A	54815	0001568 // blood vessel	0005634 // nucleus //	0003700 // sequence-	chr19:19496641-19619736
11740565_at	-0.43461227	-0.2898245	-0.42374277	0.2898245	0.39198303	0.38168907	0.001085089	0.00020279	-1.666967	-0.7372254	1.6669668	down	PNLIPRP3	119548	0006629 // lipid metabolic	0005576 // extracellular	0003824 // catalytic activity	chr10:118187381-
11720230_at	-0.28611708	-0.4500265	-0.4252677	0.28611755	0.53734636	0.35945797	0.001898572	0.00098351	-1.718851	-0.7814444	1.7188509	down	TES	26136	0008285 // negative	0005634 // nucleus //	0008270 // zinc ion binding	chr7:115850579-
11758857_at	-0.59662294	-0.4026351	-0.74124765	0.41356707	0.4160838	0.4026351	0.001429948	0.00054224	-1.987467	-0.99093056	1.9874666	down	RAB2A	5862	0006184 // GTP catabolic	0000139 // Golgi membrane	0000166 // nucleotide	chr8:61429476-61536191
11720421_at	0.38313103	0.36194754	0.29987335	-0.31115007	-0.6527004	-0.29987335	0.00398403	0.00289421	1.704748	0.76955855	1.704748	up	AFF4	27125	0006351 // transcription,	0005634 // nucleus //	0003700 // sequence-	chr5:132211075-
11739302_a_at	-0.42390776	-0.48421383	-0.4695959	0.5285597	0.4346094	0.42390728	0.000566972	1.6938E-05	-1.894212	-0.92159796	1.8942122	down	FZD6	8323	0001525 // angiogenesis //	0005737 // cytoplasm // not	0004871 // signal	chr8:104311058-
11759554_at	-0.28053522	-0.4388318	-0.554894	0.28053522	0.3860774	0.29346418	0.001901125	0.00098595	-1.675718	-0.7447792	1.6757178	down	PAPOLA	10914	0000398 // nuclear mRNA	0005634 // nucleus //	0000166 // nucleotide	chr14:96968726-97001227
11716018_s_at	-0.43225956	-0.37766075	-0.5745573	0.447402	0.3800726	0.37766075	0.001030364	0.00016417	-1.819074	-0.86320436	1.8190742	down	RAB31	11031	0006184 // GTP catabolic	0005769 // early endosome	0000166 // nucleotide	chr18:9707962-9862548 (+)
11742433_a_at	0.2702427	0.29637814	0.19287443	-0.46103096	-0.4000845	-0.19287491	0.003286277	0.0022446	1.5204467	0.6044952	1.5204467	up	ABCC9	10060	0006200 // ATP catabolic	0005886 // plasma	0000166 // nucleotide	chr12:21958107-22089628
11748362_s_at	-0.29972458	-0.35571146	-0.55978966	0.3668623	0.29972506	0.46205568	0.001973392	0.00105403	-1.718666	-0.7812896	1.7186664	down	ABCC3	8714	0006200 // ATP catabolic	0005624 // membrane	0000166 // nucleotide	chr17:48712261-48769062
11746159_a_at	0.47937965	0.34474897	0.27253485	-0.3155117	-0.2839532	-0.27253485	0.001375919	0.00044974	1.5759492	0.65622103	1.5759492	up	PBX3	5090	0002087 // regulation of	0005634 // nucleus //	0003677 // DNA binding //	chr9:128677962-
11734823_a_at	0.37297773	0.36703348	0.3013606	-0.44021797	-0.40784073	-0.3013606	0.000894235	0.00010715	1.6589423	0.7302637	1.6589423	up	APBB2	323	0001764 // neuron migration	0005622 // intracellular //	0001540 // beta-amyloid	chr4:40812044-41216635 (-
11744923_s_at	0.38001537	0.2542343	0.28131676	-0.4296441	-0.33520985	-0.2542348	0.001422641	0.00052878	1.5636147	0.64488506	1.5636147	up	RBM15	64783	0001569 // patterning of	0005634 // nucleus //	0000166 // nucleotide	chr1:110881944-
11725074_a_at	0.30364323	0.36873722	0.25775242	-0.31976223	-0.25775242	-0.33832073	0.000894235	0.00010809	1.5319006	0.6153227	1.5319006	up	FAM126A	84668	0007165 // signal	0005737 // cytoplasm //	0004871 // signal	chr7:22980877-23053814 (-
11721677_a_at	-0.278378	-0.43220615	-0.5644069	0.28340483	0.30431223	0.278378	0.001928148	0.00100649	-1.64	-0.71369535	1.6399995	down	RBM17	84991	0006397 // mRNA	0005634 // nucleus //	0000166 // nucleotide	chr10:6131231-6159420 (+)
11728828_at	-0.19099951	-0.25996637	-0.26886702	0.51112795	0.19099903	0.651248	0.008727141	0.00752857	-1.61448	-0.6910693	1.6144797	down	F2RL1	2150	0002286 // T cell activation	0005794 // Golgi apparatus	0001965 // G-protein alpha-	chr5:76114832-76131140
11758907_at	-0.3001356	-0.46110535	-0.29061317	0.29061317	0.37529707	0.31543636	0.001296612	0.00036755	-1.599625	-0.67773354	1.5996248	down	ZNF827	152485	0006351 // transcription,	0005622 // intracellular //	0003676 // nucleic acid	chr4:146678779-
11759114_at	-0.387424	-0.69571495	-0.70163774	0.41643095	0.39578676	0.38742447	0.001562753	0.00067035	-1.992813	-0.9948063	1.992813	down	LOC100506548	100506548 ///	0000184 // nuclear-	0005622 // intracellular //	0003723 // RNA binding //	chr5:40825363-40835314 (-
11744506_a_at	-0.5337658	-0.24235821	-0.36460066	0.25454617	0.30454063	0.24235773	0.002719571	0.00171181	-1.566332	-0.6473897	1.5663316	down	KLHL3	26249	0016567 // protein	0005737 // cytoplasm //	0003779 // actin binding //	chr5:136953188-
11757503_s_at	-0.3565979	-0.26957607	-0.48972988	0.32007694	0.26957607	0.3002615	0.001420569	0.00052452	-1.589536	-0.6686061	1.5895364	down	PNN	5411	0000398 // nuclear mRNA	0005634 // nucleus //	0003677 // DNA binding //	chr14:39651574-39652143
11745336_a_at	0.36502123	0.47571754	0.3003149	-0.60077477	-0.40379715	-0.3003149	0.002291941	0.00132436	1.7596804	0.81531346	1.7596804	up	DMXL1	1657				chr5:118406899-
11756328_s_at	-0.37389946	-0.21722555	-0.45909548	0.33474588	0.21722555	0.35666466	0.002390926	0.0014119	-1.572383	-0.6529522	1.5723825	down	TARDBP	23435	0006351 // transcription,	0005634 // nucleus //	0000166 // nucleotide	chr1:11085096-11085548
11748733_a_at	0.29441452	0.3256607	0.30136013	-0.29904175	-0.39079762	-0.29441452	0.000677108	4.1818E-05	1.553185	0.6352297	1.553185	up	RABGEF1	154881 /// 27342	0006810 // transport //	0005737 // cytoplasm //	0003677 // DNA binding //	chr7:66220261-66274563
11754907_a_at	0.44990826	0.50632143	0.1646018	-0.23614073	-0.1646018	-0.2478857	0.006812942	0.00563518	1.5050589	0.5898199	1.5050589	up	RNF38	152006			0008270 // zinc ion binding	chr9:36339577-36400871 (-
11734695_a_at	-0.48824883	-0.508338	-0.56357	0.5247717	0.5907488	0.48824883	0.000534553	9.5166E-06	-2.077203	-1.054642	2.0772026	down	PPP4C	5531	0000226 // microtubule	0005634 // nucleus // non-	0004704 // NF-kappaB-	chr16:30087317-30096665
11743172_a_at	0.5686779	0.5079098	0.5942154	-0.6168041	-0.5079098	-0.60724545	0.000556561	1.2518E-05	2.1950507	1.1342542	2.1950507	up	SLCO4A1	28231	0006810 // transport //	0005886 // plasma	0005215 // transporter	chr20:61273794-61303974
11751921_s_at	-0.34371758	-0.27630424	-0.32650948	0.27630424	0.34041405	0.3412428	0.000594544	2.7665E-05	-1.552756	-0.6348308	1.5527556	down	AHR	196	0000122 // negative	0005634 // nucleus //	0003677 // DNA binding //	chr7:17338357-17385767
11736478_a_at	0.3119359	0.2729144	0.34407854	-0.37302446	-0.3128972	-0.2729144	0.000744977	6.0405E-05	1.5467659	0.62925494	1.5467659	up	ELL2	22936	0006351 // transcription,	0005634 // nucleus //	0003746 // translation	chr5:95220804-95297982 (-
11736010_at	-0.33205795	-0.38643694	-0.54348946	0.33205795	0.3744521	0.41472435	0.001193643	0.00030228	-1.734363	-0.7944063	1.7343634	down	RSL1D1	26156	0006412 // translation //	0005622 // intracellular //	0003723 // RNA binding //	chr16:11927703-11945442
11736583_a_at	0.5728111	0.3572626	0.36569786	-0.36195707	-0.3572626	-0.5122318	0.001520065	0.00063486	1.79304	0.8424077	1.79304	up	TRIM36	55521	0007340 // acrosome	0001669 // acrosomal	0008270 // zinc ion binding	chr5:114460477-
11737794_a_at	0.36851835	0.3660493	0.33504725	-0.33504725	-0.4419427	-0.39795065	0.000581859	2.1974E-05	1.6796786	0.74818516	1.6796786	up	FGFR1OP	11116	0000086 // G2/M transition	0005634 // nucleus //	0005515 // protein binding //	chr6:167412815-
11732299_at	-0.37585115	-0.40290356	-0.42369843	0.37585115	0.41766644	0.38346577	0.000325438	1.9273E-06	-1.732849	-0.79314554	1.7328485	down	KIAA1715	80856	0007275 // multicellular	0016020 // membrane //		chr2:176788482-
11729271_a_at	0.35728645	0.36369514	0.25895786	-0.25895786	-0.3329544	-0.40450478	0.001140264	0.00025886	1.578753	0.65878546	1.578753	up	ASB1	51665	0007275 // multicellular	0005622 // intracellular //		chr2:239335206-
11736057_s_at	-0.25988483	-0.29109097	-0.34274483	0.29931355	0.25988483	0.518754	0.002426724	0.00144536	-1.577046	-0.6572243	1.5770456	down	C10orf32	119032				chr10:104613966-
11753586_s_at	0.246459	0.4024105	0.43575096	-0.25708866	-0.33800602	-0.246459	0.001486619	0.00059322	1.5605538	0.6420581	1.5605538	up	CXADRP2	1525 /// 440224	0007005 // mitochondrion	0001669 // acrosomal	0004872 // receptor activity	chr21:18885447-18937933
11753586_s_at	0.246459	0.4024105	0.43575096	-0.25708866	-0.33800602	-0.246459	0.001486619	0.00059322	1.5605538	0.6420581	1.5605538	up	CXADRP3	1525 /// 440224	0007005 // mitochondrion	0001669 // acrosomal	0004872 // receptor activity	chr21:18885447-18937933
11730048_at	-0.31802082	-0.4779415	-0.4771061	0.31802082	0.32506752	0.41091728	0.001103597	0.00022001	-1.71201	-0.77569133	1.7120103	down	SLITRK5	26050	0007409 // axonogenesis //	0016020 // membrane //		chr13:88324538-88331868
11747210_a_at	-0.27486992	-0.23350143	-0.36620235	0.2729702	0.46943855	0.23350143	0.002747926	0.00173663	-1.5335	-0.61682796	1.5334998	down	SRI	6717	0001508 // regulation of	0005622 // intracellular //	0005102 // receptor binding	chr7:87834972-87849351 (-
11722963_a_at	-0.37321377	-0.32040405	-0.46637917	0.3519268	0.41473484	0.32040405	0.000935536	0.000124	-1.680652	-0.74902093	1.6806519	down	COPZ2	51226	0006810 // transport //	0000139 // Golgi membrane		chr17:46103532-46115152
11728763_x_at	-0.32794428	-0.38157463	-0.32205677	0.32205677	0.39032602	0.5581541	0.001378478	0.00045469	-1.702165	-0.7673709	1.702165	down	GAL3ST4	79690	0006790 // sulfur compound	0005624 // membrane	0001733 //	chr7:99756866-99766373 (-
11715484_a_at	0.2777195	0.3399744	0.31922436	-0.5031538	-0.2777195	-0.39952278	0.00138566	0.00047743	1.6310166	0.70577145	1.6310166	up	MCL1	4170	0001709 // cell fate	0005634 // nucleus //	0005515 // protein binding //	chr1:150547031-
11730770_at	-0.2989993	-0.251976	-0.44431877	0.251976	0.26321793	0.35792065	0.001642212	0.00074238	-1.539864	-0.62280285	1.539864	down	NAPG	8774	0006461 // protein complex	0005622 // intracellular //	0005515 // protein binding //	chr18:10525853-10552766
11721446_a_at	0.40656757	0.3366499	0.34191132	-0.3869133	-0.3574934	-0.3366499	0.000555711	1.1273E-05	1.6495377	0.72206175	1.6495377	up	SNX4	8723	0006810 // transport //	0005737 // cytoplasm //	0005515 // protein binding //	chr3:125165493-
11735066_at	-0.5407038	-0.4165082	-0.5927887	0.41650772	0.4482746	0.6521044	0.001284222	0.00034931	-2.031148	-1.0222957	2.0311484	down	ITGB3	3690	0001934 // positive	0005886 // plasma	0003756 // protein disulfide	chr17:45331192-45390501
11720293_s_at	0.48488092	0.4862671	0.4608388	-0.5864568	-0.7842417	-0.4608388	0.00123664	0.00032503	2.1255577	1.0878414	2.1255577	up	LIN28B	389421	0006355 // regulation of	0005634 // nucleus //	0003676 // nucleic acid	chr6:105404922-
11740482_a_at	0.5903921	0.2017374	0.29914808	-0.22587156	-0.20173693	-0.26562548	0.008524494	0.00732731	1.5103022	0.5948372	1.5103022	up	PAQR5	54852	0007275 // multicellular	0016020 // membrane //	0004872 // receptor activity	chr15:69606741-69700089
11757917_s_at	-0.30876827	-0.21043348	-0.55732775	0.26688814	0.22899675	0.21043348	0.005882154	0.00472928	-1.509722	-0.5942826	1.5097218	down	CNOT6L	246175	0000288 // nuclear-	0005737 // cytoplasm //	0004518 // nuclease activity	chr4:78634540-78635739 (-
11715778_a_at	-0.26840973	-0.32301235	-0.45174217	0.26840973	0.34627342	0.30275917	0.001298686	0.00037359	-1.573018	-0.65353554	1.5730184	down	MTPN	136319 ///	0006417 // regulation of	0005634 // nucleus //		chr7:135611507-
11719551_s_at	-0.32286167	-0.36200905	-0.5965576	0.32286167	0.4049635	0.4902172	0.001974541	0.0010568	-1.78158	-0.8331569	1.7815795	down	RHOBTB1	9886	0007264 // small GTPase	0005622 // intracellular //	0000166 // nucleotide	chr10:62629199-62704084
11738142_a_at	0.3083105	0.3516779	0.3185196	-0.3083105	-0.31062603	-0.37580252	0.000566128	1.3853E-05	1.5776192	0.65774906	1.5776192	up	SSX2IP	117178	0007155 // cell adhesion //	0005634 // nucleus //	0005515 // protein binding //	chr1:85109598-85156180 (-
11728761_a_at	-0.29148388	-0.26009083	-0.50140095	0.26009083	0.3951378	0.38584995	0.0023001	0.00133102	-1.622275	-0.6980181	1.6222746	down	RUNX1	100506403 ///	0001501 // skeletal system	0005604 // basement	0000975 // regulatory region	chr21:36160098-36261004
11722287_a_at	-0.3632264	-0.27671528	-0.49051285	0.27671528	0.28897476	0.34554577	0.00139453	0.00048765	-1.602766	-0.68056345	1.6027656	down	SLC30A7	148867	0006810 // transport //	0000139 // Golgi membrane	0008324 // cation	chr1:101361645-
11717196_a_at	-0.60081196	-0.5714264	-0.73032045	0.68139076	0.7432518	0.57142544	0.00071607	4.9689E-05	-2.461508	-1.2995422	2.4615076	down	MEST	4232	0007498 // mesoderm	0005783 // endoplasmic	0003824 // catalytic activity	chr7:130131912-
11725599_at	-0.25097847	-0.32078028	-0.3207116	0.37243557	0.250978	0.41710472	0.001192468	0.00029963	-1.563013	-0.64432955	1.5630127	down	BEAN1	146227	0008219 // cell death //	0016020 // membrane //		chr16:66503501-66516740
11739435_a_at	-0.5062728	-0.3847847	-0.30479002	0.30479002	0.3372574	0.31601858	0.001152694	0.00026769	-1.644867	-0.7179712	1.6448673	down	FN1	2335	0001525 // angiogenesis //	0005576 // extracellular	0002020 // protease binding	chr2:216225180-
11754649_s_at	-0.54649925	-0.606277	-0.68281794	0.78633356	0.54649925	0.71474504	0.000874123	9.0962E-05	-2.452734	-1.2943907	2.4527338	down	ITK	3702	0001816 // cytokine	0005622 // intracellular //	0000166 // nucleotide	chr5:156668600-
11724848_a_at	-0.21265888	-0.27367687	-0.7763033	0.21265936	0.28074694	0.25898075	0.021317314	0.02016308	-1.592922	-0.6716754	1.5929217	down	DIXDC1	85458	0007049 // cell cycle //	0005622 // intracellular //	0003779 // actin binding //	chr11:111807926-

11747194_a_at	0.55005646	0.56110907	0.40667152	-0.40667152	-0.53904057	-0.5763254	0.000961404	0.00014457	2.0185113	1.0132916	2.0185113	up	EVC	2121	0001501 // skeletal system	0005737 // cytoplasm //		chr4:5712941-5758089 (+)
11760321_at	-0.23874187	-0.29469156	-0.27448988	0.33154345	0.4084239	0.23874187	0.00123664	0.00032455	-1.511043	-0.59554416	1.5110425	down	ZMYM6	9204	0007010 // cytoskeleton	0005634 // nucleus //	0003676 // nucleic acid	chr1:35482849-35497569 (-
11715458_a_at	-0.31828308	-0.37706757	-0.4286995	0.35768032	0.33754063	0.31828308	0.000611709	3.0158E-05	-1.638662	-0.7125181	1.6386617	down	COX8A	1351	0006091 // generation of	0005739 // mitochondrion //	0004129 // cytochrome-c	chr11:63742063-63744015
11726438_a_at	0.36394358	0.2206955	0.2941532	-0.29930544	-0.2206955	-0.42206383	0.001988532	0.00106829	1.5230384	0.6069523	1.5230384	up	MALT1	10892	0001923 // B-1 B cell	0005634 // nucleus //	0002020 // protease binding	chr18:56338617-56417212
11724345_at	0.28308153	0.5041199	0.3277645	-0.33232784	-0.39977884	-0.28308153	0.001602795	0.00071167	1.6358625	0.7100514	1.6358625	up	ITGA4	3676	0001974 // blood vessel	0005886 // plasma	0001968 // fibronectin	chr2:182321458-
11722064_at	-0.42402458	-0.37199235	-0.5311544	0.56269026	0.3719926	0.6382618	0.001381386	0.00046465	-1.954372	-0.9667053	1.9543723	down	REEP3	221035		0016020 // membrane //		chr10:65281112-65384883
11748733_a_at	0.29441452	0.3256607	0.30136013	-0.29904175	-0.39079762	-0.29441452	0.000677108	4.1818E-05	1.553185	0.6352297	1.553185	up	KCTD7	154881 /// 27342	0006810 // transport //	0005737 // cytoplasm //	0003677 // DNA binding //	chr7:66220261-66274563
11752928_x_at	-0.3949275	-0.6189332	-0.6156721	0.508399	0.4872694	0.39492798	0.001118638	0.00025174	-2.009323	-1.0067097	2.0093234	down	PPIA	5478	0000413 // protein peptidyl-	0005576 // extracellular	0003755 // peptidyl-prolyl	chr7:44836279-44841999
11759440_at	0.54512405	0.33530426	0.422966	-0.563077	-0.5582237	-0.33530426	0.001572978	0.00068068	1.892115	0.9199998	1.892115	up	GLS	2744	0002087 // regulation of	0005737 // cytoplasm //	0004359 // glutaminase	chr2:191745546-
11758736_at	-0.45636702	-0.37587786	-0.5789223	0.38445234	0.37587833	0.4623146	0.001044088	0.00017515	-1.837746	-0.87793744	1.837746	down	PSMC2	5701	0000075 // cell cycle	0000502 // proteasome	0000166 // nucleotide	chr7:102985360-
11759630_at	-0.35144424	-0.49708462	-0.39576054	0.593205	0.35144424	0.83943176	0.003418416	0.00237438	-2.013153	-1.0094568	2.0131528	down	GPATCH2	55105	0010923 // negative		0003676 // nucleic acid	chr1:217781530-
11758629_s_at	-0.23794365	-0.42170048	-0.5543299	0.23794365	0.3789158	0.3058653	0.003115956	0.0020817	-1.638338	-0.7122329	1.6383379	down	SPTBN1	6711	0000281 // cytokinesis after	0005634 // nucleus //	0003779 // actin binding //	chr2:54889100-54889445
11755800_a_at	-0.61021566	-0.4624691	-0.52517986	0.4624691	0.51695204	0.67568016	0.000970773	0.00014729	-2.120379	-1.084322	2.1203787	down	CCDC30	728621				chr1:42929024-43119852
11725296_a_at	0.38846684	0.2393961	0.3041787	-0.29779625	-0.2393961	-0.30807638	0.001118466	0.00025095	1.5077913	0.5924368	1.5077913	up	PHF7	51533		0005634 // nucleus //	0008270 // zinc ion binding	chr3:52444439-52457657
11721907_at	-0.50050163	-0.46069527	-0.5807781	0.5221987	0.47005463	0.46069527	0.000566185	1.5553E-05	-1.997656	-0.9983078	1.9976555	down	SGPP1	81537	0006665 // sphingolipid	0005624 // membrane	0003824 // catalytic activity	chr14:64150933-64194834
11736111_a_at	0.35241795	0.42479563	0.27008915	-0.36115313	-0.2726593	-0.27008915	0.001152694	0.00026834	1.5696049	0.6504015	1.5696049	up	ARHGAP18	93663	0007165 // signal	0005622 // intracellular //	0005096 // GTPase	chr6:129897289-
11728761_a_at	-0.29148388	-0.26009083	-0.50140095	0.26009083	0.3951378	0.38584995	0.0023001	0.00133102	-1.622275	-0.6980181	1.6222746	down	LOC100506403	100506403 ///	0001501 // skeletal system	0005604 // basement	0000975 // regulatory region	chr21:36160098-36261004
11755822_a_at	-0.36789227	-0.39867163	-0.3211441	0.37755013	0.35923576	0.3211441	0.000566128	1.395E-05	-1.641725	-0.7152127	1.6417253	down	TDO2	6999	0006568 // tryptophan	0005625 // soluble fraction //	0004833 // tryptophan 2,3-	chr4:156826972-
11729150_at	-0.4186945	-0.3030901	-0.41398573	0.3030901	0.3906746	0.57963943	0.001765377	0.00086925	-1.744796	-0.80305815	1.7447957	down	PIGM	93183	0006501 // C-terminal	0005783 // endoplasmic	0000030 //	chr1:159997461-
11725220_a_at	0.31820345	0.33080864	0.3160839	-0.31608438	-0.33213043	-0.33386374	0.000290487	9.5441E-08	1.5681442	0.6490582	1.5681442	up	SLK	9748	0006468 // protein	0005737 // cytoplasm //	0000166 // nucleotide	chr10:105727469-
11741759_a_at	0.5082197	0.45106792	0.3692441	-0.43516636	-0.5878072	-0.3692441	0.001170338	0.00028639	1.8750336	0.9069165	1.8750336	up	PAK3	5063	0000165 // MAPK cascade	0005737 // cytoplasm //	0000166 // nucleotide	chrX:110187468-
11722759_a_at	0.37555504	0.48474932	0.2775979	-0.35997486	-0.385314	-0.27759838	0.001375919	0.00045203	1.6474825	0.7202631	1.6474825	up	CAMSAP2	23271		0005737 // cytoplasm //	0005515 // protein binding //	chr1:200708685-
11717765_a_at	-0.3160391	-0.37854004	-0.50298786	0.34588814	0.3160391	0.38411522	0.001100865	0.0002139	-1.679312	-0.74786985	1.6793115	down	MGLL	11343	0006629 // lipid metabolic	0005886 // plasma	0004091 //	chr3:127407908-
11749518_a_at	-0.6409688	-0.355546	-0.48163986	0.37312984	0.355546	0.39406347	0.00138566	0.00048196	-1.823822	-0.8669647	1.8238217	down	SRD5A1	6715	0001655 // urogenital	0005737 // cytoplasm //	0003865 // 3-oxo-5-alpha-	chr5:6633539-6668962 (+)
11719653_at	-0.3208847	-0.464808	-0.51979303	0.32088518	0.348145	0.36008787	0.001093891	0.00020962	-1.714991	-0.7782013	1.7149913	down	SV2A	9900	0006810 // transport //	0005737 // cytoplasm //	0004872 // receptor activity	chr1:149874869-
11729406_a_at	-0.37531996	-0.4038515	-0.43318653	0.37531996	0.5768547	0.5224347	0.000954117	0.0001375	-1.860455	-0.8956558	1.8604554	down	IFNGR1	3459	0007165 // signal	0005886 // plasma	0004872 // receptor activity	chr6:137518620-
11753359_x_at	0.31705952	0.22146034	0.30722713	-0.22146034	-0.49363375	-0.34962177	0.002646563	0.00164347	1.5548991	0.6368209	1.5548991	up	PIGA	5277	0006501 // C-terminal	0000506 //	0005515 // protein binding //	chrX:15339446-15353666
11739802_at	0.50481653	0.36520147	0.2535286	-0.33247185	-0.37467527	-0.2535286	0.001932381	0.00101	1.6185936	0.6947408	1.6185936	up	VCPIP1	80124	0006508 // proteolysis //	0005737 // cytoplasm //	0004843 // ubiquitin-specific	chr8:67540721-67579482 (-
11759828_s_at	-0.33269644	-0.29199505	-0.2711401	0.2711401	0.31285763	0.28520298	0.000555711	1.1283E-05	-1.50352	-0.5883441	1.50352	down	SP100	6672	0000122 // negative	0005634 // nucleus //	0003677 // DNA binding //	chr2:231280933-
11746094_a_at	0.2821846	0.34828377	0.30440617	-0.29463577	-0.2821846	-0.31630707	0.000534553	9.7986E-06	1.5255548	0.609334	1.5255548	up	MPP4	58538		0005737 // cytoplasm //		chr2:202509599-
11727302_a_at	0.3579011	0.32910872	0.25178576	-0.25178576	-0.33193445	-0.5270791	0.002449153	0.00146286	1.6056955	0.68319833	1.6056955	up	TMEM71	137835		0016020 // membrane //		chr8:133722190-
11715646_a_at	-0.36790657	-0.28576946	-0.295928	0.28576946	0.34010887	0.29377556	0.000646508	3.5735E-05	-1.540166	-0.623086	1.5401661	down	ADIPOR1	51094	0006629 // lipid metabolic	0005886 // plasma	0004872 // receptor activity	chr1:202909957-
11719554_at	-0.3307457	-0.3407793	-0.36127234	0.3307457	0.41857338	0.48987865	0.000858566	8.5953E-05	-1.690361	-0.7573317	1.6903614	down	CTSS	1520	0002250 // adaptive immune	0005576 // extracellular	0004197 // cysteine-type	chr1:150703020-
11732408_at	0.31973505	0.4197135	0.2827754	-0.2827754	-0.32163668	-0.3458743	0.000936118	0.00012587	1.5773506	0.6575034	1.5773506	up	LMBRD2	92255		0016020 // membrane //		chr5:36103064-36152063 (-
11741373_a_at	0.40763092	0.48892546	0.1820116	-0.1820116	-0.2711463	-0.27105093	0.004470787	0.00338524	1.5166893	0.6009256	1.5166893	up	HIPK3	10114	0006351 // transcription,	0005634 // nucleus //	0000166 // nucleotide	chr11:33279167-33375939
11732203_at	0.33692455	0.49132824	0.44857502	-0.40054703	-0.41832066	-0.33692455	0.000894235	0.00010056	1.754273	0.8108733	1.754273	up	KPNA5	3841	0006606 // protein import	0005634 // nucleus //	0005515 // protein binding //	chr6:117002312-
11731465_a_at	-0.33244228	-0.25395584	-0.4264679	0.25395584	0.2920122	0.25844336	0.001183059	0.00029423	-1.521779	-0.60575914	1.5217793	down	CTSC	1075	0001913 // T cell mediated	0005764 // lysosome //	0004252 // serine-type	chr11:88053980-88070940
11717865_at	-0.26357841	-0.31540728	-0.4320221	0.39412022	0.32833958	0.2635789	0.001358874	0.00043893	-1.586318	-0.6656822	1.5863183	down	PSME4	23198	0000075 // cell cycle	0000502 // proteasome		chr2:54091203-54197977 (-
11734668_at	-0.24170494	-0.33511257	-0.47094393	0.31476784	0.24170494	0.2900324	0.001735899	0.00083311	-1.549091	-0.63142216	1.5490913	down	KPNA1	3836	0000018 // regulation of	0005634 // nucleus //	0005515 // protein binding //	chr3:122140747-
11739581_at	0.2784481	0.28519726	0.2984953	-0.2784481	-0.40337563	-0.32171917	0.00080118	7.42E-05	1.5388947	0.6218945	1.5388947	up	PRKD3	23683	0006468 // protein	0005622 // intracellular //	0000166 // nucleotide	chr2:37477644-37551513 (-
11739882_a_at	-0.29047394	-0.3603711	-0.42495203	0.29047346	0.29634047	0.5395856	0.002230716	0.001272	-1.66332	-0.73406553	1.6633197	down	GLIS3	169792	0000122 // negative	0005622 // intracellular //	0003676 // nucleic acid	chr9:3824127-4300035 (-)
11732111_a_at	-0.28496742	-0.40597153	-0.4307251	0.38872337	0.29045868	0.28496742	0.001112119	0.0002462	-1.619189	-0.69527113	1.6191887	down	TNFRSF21	27242	0006915 // apoptotic	0005737 // cytoplasm //	0004872 // receptor activity	chr6:47199268-47277680 (-
11715796_s_at	-0.86091757	-0.8056121	-1.241715	1.0196328	0.8056121	1.0664406	0.00114834	0.00026166	-3.819305	-1.93331	3.8193047	down	LUM	4060	0007601 // visual perception	0005576 // extracellular	0005201 // extracellular	chr12:91497081-91505608
11724545_at	0.26431656	0.2940297	0.26302767	-0.37761402	-0.5164404	-0.2630272	0.001772697	0.00087458	1.5795189	0.6594852	1.5795189	up	HCFC2	29915	0000122 // negative	0005634 // nucleus //	0003713 // transcription	chr12:104458234-
11721524_s_at	-0.71041393	-0.31648064	-0.527823	0.32099676	0.3164811	0.34735966	0.002779174	0.00176892	-1.798156	-0.8465184	1.7981563	down	ZNF706	51123		0005622 // intracellular //	0008270 // zinc ion binding	chr8:102209271-
11759818_a_at	-0.28425598	-0.361331	-0.3496647	0.31738853	0.34976435	0.28425598	0.000609305	2.9024E-05	-1.567958	-0.6488868	1.5679579	down	DDHD2	23259	0016042 // lipid catabolic	0005737 // cytoplasm //	0016787 // hydrolase	chr8:38089470-38120282
11718401_a_at	-0.7541466	-0.69719934	-1.0464792	0.81264734	0.69719887	0.7532029	0.000978684	0.00015026	-3.004153	-1.5869582	3.0041528	down	NUDT21	11051	0000398 // nuclear mRNA	0005634 // nucleus //	0003723 // RNA binding //	chr16:56463046-56485269
11725333_at	0.32898235	0.33253574	0.29749966	-0.33264828	-0.29749966	-0.4015131	0.000637897	3.4037E-05	1.5839859	0.66355956	1.5839859	up	MZT1	440145	0033566 // gamma-tubulin	0005737 // cytoplasm //	0005515 // protein binding //	chr13:73282504-73302001
11733375_a_at	0.3311782	0.3874035	0.23514748	-0.55593634	-0.32870007	-0.23514748	0.003850937	0.0027682	1.6145936	0.69117105	1.6145936	up	MSI2	124540	0048864 // stem cell	0005737 // cytoplasm //	0000166 // nucleotide	chr17:55333894-55757299
11739469_at	0.3235569	0.25339222	0.29036188	-0.26829863	-0.5030384	-0.25339222	0.002634147	0.00163501	1.5482947	0.6306801	1.5482947	up	TMEM87B	84910		0016020 // membrane //		chr2:112812799-
11733293_at	-0.4208727	-0.40242195	-0.37021828	0.37021828	0.41078234	0.47436905	0.000566972	1.7295E-05	-1.760877	-0.8162942	1.760877	down	PHF20L1	51105			0008270 // zinc ion binding	chr8:133787576-
11715739_s_at	-0.27989864	-0.3585291	-0.26938295	0.35755157	0.26938248	0.3721323	0.000922398	0.00011836	-1.553611	-0.6356257	1.5536114	down	PPAP2B	8613	0001568 // blood vessel	0005789 // endoplasmic	0003824 // catalytic activity	chr1:56960425-57109300 (-
11735431_x_at	-0.34406328	-0.3095932	-0.3793497	0.34129286	0.33021307	0.30959272	0.000534553	7.091E-06	-1.592583	-0.6713683	1.5925827	down	TMBIM4	51643	0006915 // apoptotic	0005794 // Golgi apparatus	0005515 // protein binding //	chr12:66530716-66563807
11720817_a_at	-0.3117876	-0.35388088	-0.32503605	0.31472588	0.37121868	0.3117876	0.000534553	8.6113E-06	-1.583166	-0.66281223	1.5831656	down	BAALC	79870		0005634 // nucleus //		chr8:104152602-
11738232_a_at	0.28388262	0.29755354	0.27887154	-0.37207127	-0.27887154	-0.30063963	0.000611709	3.0127E-05	1.5198863	0.6039634	1.5198863	up	CENPI	2491	0000087 // M phase of	0000775 // chromosome,	0005515 // protein binding //	chrX:100353156-
11722083_s_at	-0.26496935	-0.22249508	-0.5656233	0.34339857	0.22249508	0.29195976	0.006133739	0.00495858	-1.555071	-0.6369804	1.555071	down	RUFY3	22902	0007275 // multicellular	0030175 // filopodium //		chr4:71587675-71659274
11752409_a_at	0.31564236	0.37235737	0.3311367	-0.31564236	-0.4525919	-0.34601307	0.000878822	9.239E-05	1.6370834	0.7111279	1.6370834	up	TMEM48	55706	0006810 // transport //	0005634 // nucleus //	0017056 // structural	chr1:54233309-54303940 (-
11729033_at	-0.29981613	-0.33164215	-0.43701172	0.29981613	0.31990814	0.30015087	0.000881439	9.4102E-05	-1.583132	-0.6627817	1.5831321	down	ITPRIPL2	162073		0016020 // membrane //		chr16:19125271-19132951
11723894_a_at	-0.5333786	-0.19699049	-0.4440446	0.19699049	0.28117943	0.20756578	0.005108757	0.0039778	-1.536928	-0.62004983	1.5369283	down	TEP1	7011	0000722 // telomere	0000781 // chromosome,	0000166 // nucleotide	chr14:20833825-20876624
11727445_a_at	-0.46309233	-0.22824001	-0.43698692	0.2989211	0.23904085	0.22824001	0.002186191	0.00123434	-1.549182	-0.63150704	1.5491824	down	FAM70A	55026		0016020 // membrane //		chrX:119392504-
11720855_a_at	0.4706998	0.38448286	0.34340382	-0.34340382	-0.53999376	-0.43838692	0.00111501	0.00024715	1.7902036	0.84012365	1.7902036	up	NFIA	4774	0006260 // DNA replication	0005622 // intracellular //	0003677 // DNA binding //	chr1:61547979-61928465
