# Supplementary material for: Silencing NUDT21 Attenuates the Mesenchymal Identity of Glioblastoma Cells via the NF-κB Pathway
Source: Front Mol Neurosci. 2017 Dec 19;10:420. doi: 10.3389/fnmol.2017.00420 (PMC5742174; doi:10.3389/fnmol.2017.00420)
Supplement: Supplementary file 4 [file Table_2.PDF]

Tabel S2.General Feature of Patients								
Sample No.	Age	Sex	Family history of cancer（Y/N)	History of smoking（Y/N)	History of drinking（Y/N)	Diagnosis	Tumor size ≥ 4.5cm（Y/N)	Tumor location（Supratentorial/Subtentorial）
G-0666	65	Male	No	No	No	GBM (WHO IV)	Yes	Supratentorial
G-0996	67	Male	No	No	No	GBM (WHO IV)	Yes	Supratentorial
G-0285	62	Female	No	No	No	GBM (WHO IV)	No	Supratentorial
G-0004	33	Female	No	No	No	GBM (WHO IV)	No	Supratentorial
G-0821	54	Male	No	No	No	GBM (WHO IV)	Yes	Supratentorial
G-0558	55	Female	No	No	No	GBM (WHO IV)	Yes	Supratentorial
G-0396	55	Female	No	No	No	GBM (WHO IV)	Yes	Supratentorial
G-0729	36	Male	No	Yes	No	GBM (WHO IV)	Yes	Supratentorial
G-0266	41	Female	No	No	No	GBM (WHO IV)	Yes	Supratentorial
G-0940	57	Female	No	No	No	GBM (WHO IV)	No	Supratentorial
G-0944	60	Female	No	No	No	GBM (WHO IV)	Yes	Supratentorial
G-0115	60	Male	No	No	No	GBM (WHO IV)	No	Supratentorial
G-0132	48	Male	No	Yes	Yes	GBM (WHO Ⅲ-IV)	Yes	Supratentorial
G-0839	61	Male	No	No	No	GBM (WHO IV)	Yes	Supratentorial
